# Synthesis of new pyrazolyl-2, 4-thiazolidinediones as antibacterial and antifungal agents

**DOI:** 10.1186/2191-2858-1-15

**Published:** 2011-11-08

**Authors:** Deepak K Aneja, Poonam Lohan, Sanjiv Arora, Chetan Sharma, Kamal R Aneja, Om Prakash

**Affiliations:** 1 Department of Chemistry, Kurukshetra University, Kurukshetra 136119, Haryana, India; 2 Department of Microbiology, Kurukshetra University, Kurukshetra 136119, Haryana, India; 3 Institute of Pharmaceutical Sciences, Kurukshetra University, Kurukshetra 136119, Haryana, India

**Keywords:** thiazolidine-2, 4-dione, pyrazole, Knoevenagel condensation, antibacterial activity, antifungal activity

## Abstract

**Background:**

Thiazolidine-2, 4-diones (TZDs) have become a pharmacologically important class of heterocyclic compounds since their introduction in the form of glitazones into the clinical use for the treatment of type 2 diabetes. TZDs lower the plasma glucose levels by acting as ligands for gamma peroxisome proliferators-activated receptors. In addition, this class of heterocyclic compounds possesses various other biological activities such as antihyperglycemic, antimicrobial, anti-inflammatory, anticonvulsant, insecticidal, etc. TZDs are also known for lowering the blood pressure thereby reducing the chances of heart failure and micro-albuminuria in the patients with type 2 diabetes.

**Results:**

We have described herein the synthesis of three series of compounds, namely, ethyl 2-((*Z*)-5-((3-aryl-1-phenyl-1*H*-pyrazol-4-yl)methylene)-2, 4-dioxothiazolidin-3-yl)acetates **(4)**, methyl 2-((*Z*)-5-((3-aryl-1-phenyl-1*H*-pyrazol-4-yl)methylene)-2, 4-dioxothiazolidin-3-yl)acetates **(5)**, and 2-((*Z*)-5-((3-aryl-1-phenyl-1*H*-pyrazol-4-yl)methylene)-2, 4-dioxothiazolidin-3-yl)acetic acids **(6)**. The compounds **4 **and **5 **were synthesized by Knoevenagel condensation between 3-aryl-1-phenyl-1*H*-pyrazole-4-carbaldehydes **(1) **and ethyl/methyl 2-(2, 4-dioxothiazolidin-3-yl)acetates (**3, 2) **in alcohol using piperidine as a catalyst. The resultant compounds **4 **and **5 **having ester functionality were subjected to acidic hydrolysis to obtain **6**. All the new compounds were tested for their *in vitro *antibacterial and antifungal activity.

**Conclusions:**

Knoevenagel condensation approach has offered an easy access to new compounds **4-6**. Antimicrobial evaluation of the compounds has shown that some of the compounds are associated with remarkable antifungal activity. In case of antibacterial activity, these were found to be effective against Gram-positive bacteria. However, none of the compounds were found to be effective against Gram-negative bacteria.

## 1. Background

Natural antibiotic compounds have become essential to current health care system, assisting and complementing the natural immune system against microbial pathogens. As conventional antibiotics are often abused to treat microbial infections, some microorganisms have developed tolerance to these antibiotics. Because of the appearance of antibiotic-resistant strains, the continuous development of novel efficient antibiotic agents is more crucial than ever [1-3]. So, the medical community faces a serious problem against infections caused by the pathogen bacteria and needs an effective therapy and search for novel antimicrobial agents. Synthetic organic chemistry has always been a vital part of highly integrated and multidisciplinary process of various drug developments. In this context, this study was designed to evaluate antimicrobial properties of new pyrazole derivatives containing thiazolidindiones.

Pyrazole derivatives are known to possess wide spectrum of pharmacological properties such as antibacterial [4-6], antifungal [7-9], antimicrobial [10-14], antidiabetic [15], herbicidal [16,17], antitumor [18-21], anti-anxiety [22], and as active pharmacophore in celecoxib (as COX-2 inhibitor) [23] and slidenafil citrate [24] (as cGMP specific phosphodiesterase type 5 inhibitor), etc. Pyrazoles play an essential role in biological active compounds and therefore represent an interesting template for medicinal chemistry.

On the other hand, thiazolidines are also known for their potential biological activities. The varied biological activities of rhodanines (2-thioxo-thiazolidin-4-one) and their analogs have been known from the beginning of twentieth century. Rhodanines and 2, 4-thiazolidinediones (TZDs) have become a pharmacologically important class of heterocyclic compounds since the introduction of various glitazone and epalrestat into clinical use for the treatment of type II diabetes and diabetic complications [25]. Several studies have been reported that TZDs have acquired much importance because of their diverse pharmaceutical applications such as antihyperglycemic [26], bactericidal [27], pesticidal [28], fungicidal [29], insecticidal [30], anticonvulsant [31], tuberculostatic [32], anti-inflammatory [33] etc.

Different possibilities of heterocyclic modifications with a wide spectrum of pharmacological propertiesare the most important grounds for investigation of this class of compounds. There have been many reports in literature depicting that the presence of heterocyclic moieties such as thiazole, pyrazole, flavone, chromone, sultam, and furan at fifth position proves to be more potent and efficacious than a simple aryl group [34-39]. Although there are not many TZDs fused to pyrazoles, a number of them are incorporated into a wide variety of therapeutically important compounds possessing a broad spectrum of biological activities. In a recent article, pyrazolyl-2, 4-TZDs have been reported as anti-inflammatory and neuroprotective agents.

Motivated by these findings and in continuation of our ongoing efforts endowed with the discovery of nitrogen-containing heterocycles with potential chemotherapeutic activities [8,10,40-44], we disclose here the synthesis and investigations of antimicrobial activities of new pyrazolyl-2, 4-TZD.

## 2. Results and discussion

### 2.1. Chemistry

**Scheme 1:**
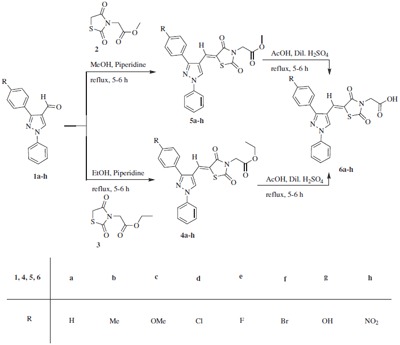
Synthesis of pyrazolyl-2, 4-TZDs (4-6).

The synthetic route for the preparation of ethyl 2-((*Z*)-5-((3-aryl-1-phenyl-1*H*-pyrazol-4-yl)methylene)-2, 4-dioxothiazolidin-3-yl)acetates **(4a-h)**, methyl 2-((*Z*)-5-((3-aryl-1-phenyl-1*H*-pyrazol-4-yl)methylene)-2, 4-dioxothiazolidin-3-yl)acetates **(5a-h)**, and 2-((*Z*)-5-((3-aryl-1-phenyl-1*H*-pyrazol-4-yl)methylene)-2, 4-dioxothiazolidin-3-yl)acetic acids **(6a-h) **has been illustrated in Scheme 1. Initially, Knoevenagel condensation was carried out with equimolar ratio of ethyl 2-(2, 4-dioxothiazolidin-3-yl)acetate **(3) **and 1, 3-diphenyl-1*H*-pyrazole-4-carbaldehyde **(1a) **in ethanol in presence of catalytic amount of piperidine by refluxing for 5-6 h. The usual work up of the reaction afforded the single product, ethyl 2-((*Z*)-2, 4-dioxo-5-((1, 3-diphenyl-1*H*-pyrazol-4-yl)methylene)thiazolidin-3-yl)acetate **(4a) **as yellow solid in 90% yield. Similar method was adopted for the preparation of **5a **in methanol. The acid hydrolysis of **4a **or **5a **in acetic acid in the presence of dilute sulfuric acid under refluxing for 5-6 h gave the desired product 2-((*Z*)-2, 4-dioxo-5-((1, 3-diphenyl-1*H*-pyrazol-4-yl)methylene)thiazolidin-3-yl)acetic acid **(6a) **in 94% yield.

All other compounds **4b-h**, **5b-h**, and **6b-h **were prepared adopting the similar methodology. The physical data of all compounds **4-6 **have been summarized in Table 1.

**Table 1:** Physical data of the compounds 4-6

Compounds	Yields (%)	Melting points (°C)
**4a**	90	223-225
**4b**	92	225-227
**4c**	91	274-276
**4d**	92	248-250
**4e**	93	237-239
**4f**	93	258-260
**4g**	94	248-250
**4h**	95	231-233
**5a**	92	225-227
**5b**	94	233-235
**5c**	91	263-265
**5d**	93	248-250
**5e**	91	233-235
**5f**	92	269-271
**5g**	90	280-282
**5h**	93	240-242
**6a**	94	294-296
**6b**	93	300-302
**6c**	94	262-264
**6d**	93	280-282
**6e**	92	304-306
**6f**	90	288-290
**6g**	94	317-319
**6h**	91	287-288

The structures of all compounds **4a-h**, **5a-h**, and **6a-h **were established by the spectral (IR, NMR {see additional files 1, 2, 3, 4, 5, 6, 7, 8, 9, 10, 11, 12, 13, 14, 15, 16, 17, 18, 19, 20, 21, 22, 23 and 24}, Mass) and elemental analysis. For example, IR spectrum of the compound **4a **exhibited characteristic absorption bands at 1736 and 1690 cm^-1 ^because of carbonyl groups of ester and TZD. The ^1^H NMR spectrum of the product **4a **(see additional files 1) showed three characteristic singlets at δ 8.213, δ 7.963, and δ 4.473 because of C(5)-H of pyrazole ring, =CH and -NCH_2_, respectively, apart from other aromatic signals. Besides these the aliphatic region also showed the characteristic quartet and triplet due to -OCH_2_CH_3 _at δ 4.248 and δ 1.301, respectively. The product **6a **was characterized by careful comparison of the IR and ^1^H NMR spectra (see additional file 17) with those of the **4a**. An important characteristic feature in ^1^H NMR spectrum of **6a **was disappearance of the triplet and quartet in the aliphatic region which was present in the spectrum of **4a**.

The starting materials 3-aryl-1-phenyl-1*H*-pyrazole-4-carbaldehydes (**1a-h**) were prepared according to literature procedure involving Vilsmeier-Haack reaction of various substituted acetophenone hydrazones using POCl_3_/DMF at 50-60°C for 4-5 h [45-47] and ethyl/methyl 2-(2, 4-dioxothiazolidin-3-yl)acetates (**3**, **2**) were prepared in multiple steps by alkylation of potassium salt of thiazolidine-2, 4-dione (TZDs) with appropriate alkyl 2-bromoacetate either in acetone at 50°C for 5 h or in KI/DMF at 90°C for 12 h [48]. The key starting material 2, 4-TZD needed for this purpose was obtained in one step from equimolar amounts of chloroacetic acid and thiourea under ice cold condition. The white precipitate of 2-imino thiazolidine-4-one obtained was then acidified and refluxed with HCl for 12 h to get white crystals of 2, 4-TZD [49].

Although geometrical isomerism (*E/Z *isomers) was possible because of restricted rotation about the exocyclic C=C bond of the pyrazolyl-2, 4-TZDs, all the derivatives prepared in this study were obtained exclusively in *Z*-form as confirmed by the analytical data. The ^1^H NMR spectra of the pyrazolyl-2, 4-TZDs (see additional files 1, 2, 3, 4, 5, 6, 7, 8, 9, 10, 11, 12, 13, 14, 15, 16, 17, 18, 19, 20, 21, 22, 23, 24) showed that the most characteristic olefinic proton =CH was deshielded more (δ = 7.3-7.6 ppm) as expected in *Z*-form, relative to the slightly shielded protons of the *E*-form (δ = 6.2-6.3 ppm, in case of various other arylidene-2, 4-TZD). This deshielding of the olefinic proton is caused by the anisotropic effect exerted by the nearby carbonyl group of the 2, 4-TZDs in *Z*-isomer. Furthermore, the *Z*-isomers are thermodynamic more stable because of intramolecular hydrogen bond that can be formed between the hydrogen bond of =CH and oxygen atom in TZD [50,51].

### 2.2. Pharmacology

#### 2.2.1. In vitro antifungal activity

All the 24 compounds were tested for their *in vitro *antifungal activity against two fungi, namely, *Aspergillus niger *and *Aspergillus flavus*. Standard antibiotic, namely, Fluconazole, was used for comparison with antifungal activity shown by compounds **4a-h**, **5a-h**, and **6a-h**. A careful analysis of percentage mycelial growth inhibition revealed that almost all the newly synthesized compounds showed comparable antifungal activity with commercial antibiotics Fluconazole as shown in Table 2.

**Table 2:** *In vitro *antifungal activity of the compounds 4-6

Compounds	Mycelial growth of inhibition (%)	
	
	*A. flavus*	*A. niger*
**4a**	54.4	60.0
**4b**	54.4	70.0
**4c**	48.8	54.4
**4d**	61.1	65.5
**4e**	67.7	61.1
**4f**	55.5	62.5
**4g**	61.1	54.4
**4h**	48.8	58.8
**5a**	62.5	55.5
**5b**	48.8	54.4
**5c**	54.4	62.5
**5d**	55.5	61.1
**5e**	57.7	55.5
**5f**	67.7	62.5
**5g**	54.4	57.7
**5h**	61.1	54.4
**6a**	61.1	62.5
**6b**	63.3	61.1
**6c**	55.5	60.0
**6d**	61.5	62.5
**6e**	65.5	62.5
**6f**	65.5	61.1
**6g**	54.4	58.8
**6h**	61.1	60.0
**Fluconazole**	77.7	81.1

Compounds **4b **and **4e **showed maximum inhibition against *A. niger *(70%) and *A. flavus *(67.7%), respectively. Eleven compounds **4d**, **4e**, **4g**, **5a**, **5h**, **6a**, **6b**, **6d**, **6e**, **6f**, and **6h **showed more than 60% inhibition against *A. flavus *in comparison to 77.7% of Fluconazole. Eleven compounds which showed more than 60% inhibition against *A. niger *are **4b**, **4d**, **4e**, **4h**, **5c, 5d, 6a**, **6b**, **6d**, **6e**, **6f**. After all, the compounds which showed more than 60% inhibition against both the pathogenic fungi are **4a**, **4e**, **6a**, **6d**, and **6e**.

#### 2.2.2. In vitro antibacterial activity

All the 24 compounds **4a-h**, **5a-h**, and **6a-h **were tested *in vitro *for their antibacterial activity against two Gram-positive bacteria, namely, *Staphylococcus aureus *(MTCC 96), *Bacillus subtillis *(MTCC 121) and two Gram-negative bacteria, namely, *Escherichia coli *(MTCC 1652), and *Pseudomonas aeruginosa *(MTCC 741) (Tables 3 and 4). Minimum inhibitory concentrations (MIC) of those compounds were determined which were showing activity in primary screening. Standard antibiotic, Ciprofloxacin, was used for comparison with antibacterial activity shown by the compounds **4a-h**, **5a-h**, and **6a-h**.

**Table 3:** *In vitro *antibacterial activity of the compounds 4-6

Compounds	Diameter of the growth of zone inhibition (mm)^a^	
	*S. aureus*	*B. subtilis*
**4a**	15.6	16.3
**4b**	16.3	15.0
**4c**	15.3	14.6
**4d**	14.3	14.6
**4e**	13.6	14.0
**4f**	16.6	17.6
**4g**	15.0	15.6
**4h**	19.0	17.0
**5a**	17.6	15.3
**5b**	18.6	16.0
**5c**	15.6	15.0
**5d**	16.3	15.6
**5e**	15.0	16.6
**5f**	16.6	16.6
**5g**	18.0	16.0
**5h**	20.0	21.0
**6a**	18.6	19.3
**6b**	18.6	19.3
**6c**	14.0	15.3
**6d**	16.6	17.3
**6e**	14.6	13.0
**6f**	13.6	14.3
**6g**	13.6	14.6
**6h**	19.0	18.0
**Ciprofloxacin**	26.0	24.0

^a^Values including diameter of the well (8 mm) are means of three replicates

**Table 4:** MIC of the compounds 4-6

Compounds	MIC (μg/mL)	
	*S. aureus*	*B. subtilis*
**4a**	128	128
**4b**	128	128
**4c**	128	128
**4d**	128	128
**4e**	128	128
**4f**	128	128
**4g**	128	128
**4h**	64	128
**5a**	128	128
**5b**	128	128
**5c**	128	128
**5d**	128	128
**5e**	128	128
**5f**	128	128
**5g**	64	128
**5h**	64	32
**6a**	64	64
**6b**	128	64
**6c**	128	128
**6d**	128	128
**6e**	128	128
**6f**	128	128
**6g**	128	128
**6h**	64	64
**Ciprofloxacin**	5	5

All compounds of the tested series showed variable antibacterial activity against Gram-positive bacteria. Three of the tested compounds **5h**, **6a**, and **6h **exhibited good antibacterial activity against Gram-positive bacteria. However, none of the compounds showed activity against Gram-negative bacteria.

In case of Gram-positive bacteria, compounds **4h**, **5b**, **5h**, **6a**, **6b**, and **6h **were found to be most effective against *S. aureus *with zone of inhibition ranging between 18.6 mm and 20.0 mm and the compounds **5h**, **6a**, and **6b **were most effective against *B. subtillis *with zone of inhibition ranging between 19.3 mm and 21.0 mm (Table 3).

In whole series, compounds **4a**, **4h**, and **5h **showed maximum antibacterial activity against *S. aureus *(MIC 64 μg/mL) and compounds **5h **(MIC 32 μg/mL), **6a **&**6h **(MIC 64 μg/mL) against *B. subtillis *(Table 4).

## 3. Conclusions

We have described herein an efficient and convenient synthesis of three series of pyrazolyl-2, 4-TZDs (**4**-**6**) by Knoevenagel condensation. All the 24 compounds synthesized were characterized by spectral and elemental analytical data and evaluated for their *in vitro *antifungal and antibacterial activities. Results of the antifungal activity were found to be comparable with the reference compound. On the other hand, antibacterial activity was best observed for Gram-positive bacteria only, none of the compounds showed activity against Gram-negative bacteria.

## 4. Experimental

### 4.1. General remarks

Melting points (mps) were taken on slides in an electrical apparatus Labindia visual melting range apparatus and are uncorrected. Calibration of melting point apparatus was done using benzoic acid as reference. IR spectra were recorded on a Perkin-Elmer 1800 FT-IR spectrophotometer. ^1^H NMR spectra (see additional files 1, 2, 3, 4, 5, 6, 7, 8, 9, 10, 11, 12, 13, 14, 15, 16, 17, 18, 19, 20, 21, 22, 23, 24) were recorded on a Bruker 300 & 400 MHz instrument using tetramethylsilane as an internal standard. Mass spectra were recorded on 2500 eV (ESI Source) using a water's Q-TOF microinstrument and elemental analysis on Perkin-Elmer 2400 instrument. All the reagents were purchased from the commercial sources and were used without further purification.

### 4.2. Preparation of ethyl 2-((Z)-5-((3-aryl-1-phenyl-1H-pyrazol-4-yl)methylene)-2, 4-dioxothiazolidin-3-yl)acetates (4a-h)

Typical procedure: A mixture of 1, 3-diphenyl-1*H*-pyrazol-4-carboxaldehyde **1a **(0.5 g, 2 mmol) and ethyl 2-(2, 4-dioxothiazolidin-3-yl)acetate **3 **(0.4 g, 2 mmol) in ethanol (20 mL) and 2-3 drops of piperidine was refluxed for 4-5 h. A solid was separated out of the reaction mixture within 15-20 min and the refluxing was continued for 4-5 h to complete the reaction. The reaction mixture was cooled to room temperature, filtered, and washed with ethanol to give the pure product **4a **(0.87 g, 90% yield).

The other derivatives **4b-h **were synthesized by adopting the similar procedure.

### 4.3. Ethyl 2-((Z)-2, 4-dioxo-5-((1, 3-diphenyl-1H-pyrazol-4-yl)methylene)thiazolidin-3-yl)acetate (4a)

IR (ν_max_, KBr) cm^-1^: 1736, 1690, 1612, 1535, 1504, 1450, 1373, 1311, 1227, 1142, 1103, 1065, 1026. ^1^H NMR (CDCl_3_, 400 MHz, δ): 8.213 (s, 1H, Pyrazolyl H), 7.963 (s, 1H, =CH), 7.817-7.795 (m, 2H, Ar H), 7.678-7.654 (m, 2H, Ar H), 7.549-7.471 (m, 5H, Ar H), 7.414-7.377 (m, 1H, Ar H), 4.473 (s, 2H, NCH_2_), 4.275-4.222 (q, 2H, -OCH_2_CH_3_), 1.319-1.283 (t, 3H, -OCH_2_CH_3_). MS (ESI+) *m/z *434 [M+H]. Anal. Found: C, 63.3; H, 4.6; N, 9.5. C_23_H_19_N_3_O_4_S requires C, 63.73; H, 4.42; N, 9.69%.

### 4.4. Ethyl 2-((Z)-2, 4-dioxo-5-((1-phenyl-3-p-tolyl-1H-pyrazol-4-yl)methylene)thiazolidin-3-yl)acetate (4b)

IR (ν_max_, KBr) cm^-1^: 1736, 1690, 1605, 1520, 1450, 1373, 1311, 1219, 1142, 1095, 1026. ^1^H NMR (DMSO-d_6_, 400 MHz, δ): 8.812 (s, 1H, Pyrazolyl H), 8.041-8.022 (m, 2H, Ar H), 7.739 (s, 1H, =CH) 7.598-7.536 (m, 4H, Ar H), 7.448-7.379 (m, 3H, Ar H), 4.480 (s, 2H, NCH_2_), 4.199-4.145 (q, 2H, -OCH_2_CH_3_), 2.405 (s, 3H, Ph CH_3_), 1.231-1.195 (t, 3H, -OCH_2_CH_3_). MS (ESI+) *m/z *448 [M+H]. Anal. Found: C, 64.0; H, 4.98; N, 9.2. C_24_H_21_N_3_O_4_S requires C, 64.41; H, 4.73, N, 9.39%.

### 4.5. Ethyl 2-((Z)-5-((3-(4-methoxyphenyl)-1-phenyl-1H-pyrazol-4-yl)methylene)-2, 4-dioxothiazolidin-3-yl)acetate (4c)

IR (ν_max_, KBr) cm^-1^: 1736, 1690, 1612, 1520, 1450, 1373, 1311, 1296, 1227, 1180, 1142, 1095, 1026, 1018. ^1^H NMR (TFA-d_1_, 400 MHz, δ): 8.483 (s, 1H, Pyrazolyl H), 7.917 (s, 1H, =CH), 7.667-7.583 (m, 7H, Ar H), 7.179-7.157 (d, 2H, Ar H, *J *= 8.8 Hz), 4.620 (s, 2H, NCH_2_), 4.345-4.291 (q, 2H, CH_2_CH_3_), 3.922 (s, 3H, Ph OCH_3_), 1.304-1.269 (t, 3H, CH_3_CH_2_). MS (ESI+) *m/z *464 [M+H]. Anal. Found: C, 61.8; H, 4.1; N, 8.6. C_24_H_21_N_3_O_5_S requires C, 62.19; H, 4.57; N, 9.07%.

### 4.6. Ethyl 2-((Z)-5-((3-(4-chlorophenyl)-1-phenyl-1H-pyrazol-4-yl)methylene)-2, 4-dioxothiazolidin-3-yl)acetate (4d)

IR (ν_max_, KBr) cm^-1^: 1736, 1690, 1612, 1528, 1443, 1373, 1311, 1227, 1142, 1095, 1011. ^1^H NMR (TFA-d_1_, 400 MHz, δ): 8.657 (s, 1H, Pyrazolyl H), 8.052 (s, 1H, =CH), 7.832-7.748 (m, 5H, Ar H), 7.748-7.724 (m, 4H, Ar H), 4.789 (s, 2H, NCH_2_), 4.515-4.462 (q, 2H, -OCH_2_CH_3_), 1.476-1.440 (t, 3H, -OCH_2_CH_3_). MS (ESI+) *m/z *454 [M+H]. Anal. Found: C, 58.6; H, 3.9; N, 8.7. C_23_H_18_ClN_3_O_4_S requires C, 59.04; H, 3.88; N, 8.98%.

### 4.7. Ethyl 2-((Z)-5-((3-(4-fluorophenyl)-1-phenyl-1H-pyrazol-4-yl)methylene)-2, 4-dioxothiazolidin-3-yl)acetate (4e)

IR (ν_max_, KBr) cm^-1^: 1736, 1697, 1612, 1512, 1450, 1373, 1311, 1234, 1142, 1095, 1026. ^1^H NMR (TFA-d_1_, 400 MHz, δ): 8.489 (s, 1H, Pyrazolyl H), 7.884 (s, 1H, =CH), 7.652-7.584 (m, 7H, Ar H), 7.290-7.247 (m, 2H, Ar H), 4.624 (s, 2H, NCH_2_), 4.351-4.297 (q, 2H, -OCH_2_CH_3_), 1.311-1.275 (t, 3H, -OCH_2_CH_3_). MS (ESI+) *m/z *437 [M+H]. Anal. Found: C, 61.0; H, 4.2; N, 9.2. C_23_H_18_FN_3_O_4_S requires C, 61.19; H, 4.02; N, 9.31%.

### 4.8. Ethyl 2-((Z)-5-((3-(4-bromophenyl)-1-phenyl-1H-pyrazol-4-yl)methylene)-2, 4-dioxothiazolidin-3-yl)acetate (4f)

IR (ν_max_, KBr) cm^-1^: 1736, 1690, 1605, 1528, 1443, 1373, 1311, 1227, 1142, 1095, 1003. ^1^H NMR (TFA-d_1_, 400 MHz, δ): 8.488 (s, 1H, Pyrazolyl H), 7.896 (s, 1H, =CH), 7.750-7.729 (m, 2H, Ar H), 7.650-7.588 (m, 5H, Ar H), 7.489-7.467 (d, 2H, Ar H, *J *= 8.8 Hz) 4.633 (s, 2H, NCH_2_), 4.359-4.305 (q, 2H, -OCH_2_CH_3_), 1.319-1.283 (t, 3H, -OCH_2_CH_3_). MS (ESI+) *m/z *497 [M+H]. Anal. Found: C, 53.7; H, 3.4; N, 8.0. C_23_H_18_BrN_3_O_4_S requires C, 53.91; H, 3.54; N, 8.20%.

### 4.9. Ethyl 2-((Z)-5-((3-(4-hydroxyphenyl)-1-phenyl-1H-pyrazol-4-yl)methylene)-2, 4-dioxothiazolidin-3-yl)acetate (4g)

IR (ν_max_, KBr) cm^-1^: 3387, 1736, 1682, 1605, 1520, 1373, 1319, 1234, 1142, 1103, 1026. ^1^H NMR (DMSO-d_6_, 400 MHz, δ): 9.850 (bs, 1H, OH), 8.773 (s, 1H, Pyrazolyl H), 8.027-8.007 (m, 2H, Ar H), 7.734 (s, 1H, =CH), 7.588-7.549 (m, 2H, Ar H), 7.474-7.452 (d, 2H, Ar H, *J *= 8.8 Hz), 7.435-7.398 (m, 1H, Ar H), 6.955-6.933 (d, 2H, Ar H, *J *= 8.8 Hz), 4.479 (s, 2H, NCH_2_), 4.199-4.146 (q, 2H, -OCH_2_CH_3_), 1.232-1.196 (t, 3H, -OCH_2_CH_3_). MS (ESI+) *m/z *435 [M+H]. Anal. Found: C, 61.3; H, 4.4; N, 9.1. C_23_H_19_N_3_O_5_S requires C, 61.46; H, 4.26; N, 9.35%.

### 4.10. Ethyl 2-((Z)-5-((3-(4-nitrophenyl)-1-phenyl-1H-pyrazol-4-yl)methylene)-2, 4-dioxothiazolidin-3-yl)acetate (4h)

IR (ν_max_, KBr) cm^-1^: 1736, 1697, 1620, 1528, 1350, 1319, 1234, 1142, 1095. ^1^H NMR (TFA-d_1_, 400 MHz, δ): 8.482-8.460 (d, 2H, Ar H, *J *= 8.8 Hz), 8.391 (s, 1H, Pyrazolyl H), 7.957 (s, 1H, =CH), 7.895-7.874 (d, 2H, Ar H, *J *= 8.4 Hz), 7.664-7.652 (m, 2H, Ar H), 7.586-7.573 (m, 3H, Ar H), 4.666 (s, 2H, NCH_2_), 4.388-4.334 (q, 2H, -OCH_2_CH_3_), 1.347-1.311 (t, 3H, -OCH_2_CH_3_). MS (ESI+) *m/z *465 [M+H]. Anal. Found: C, 57.4; H, 3.9; N, 11.6. C_23_H_18_N_4_O_6_S requires C, 57.73; H, 3.79; N, 11.71%.

### 4.11. Preparation of methyl 2-((*Z*)-5-((3-aryl-1-phenyl-1*H*-pyrazol-4-yl)methylene)-2, 4-dioxothiazolidin-3-yl)acetates (5a-h)

Typical procedure: A mixture of 1, 3-diphenyl-1*H*-pyrazol-4-carboxaldehyde **1a **(0.5 g, 2 mmol) and methyl 2-(2, 4-dioxothiazolidin-3-yl)acetate **2 **(0.38 g, 2 mmol) in methanol (20 ml) and 2-3 drops of piperidine was refluxed 4-5 h. A solid was separated out of the reaction mixture within 15-20 min and the refluxing was continued for 4-5 h to complete the reaction. The reaction mixture was cooled to room temperature, filtered and washed with methanol to give the pure product **5a **(0.84 g, 92% yield).

The other derivatives **5b-h **were synthesized by adopting the similar procedure.

### 4.12. Methyl 2-((Z)-2, 4-dioxo-5-((1, 3-diphenyl-1H-pyrazol-4-yl)methylene)thiazolidin-3-yl)acetate (5a)

IR (ν_max_, KBr) cm^-1^: 1744, 1690, 1605, 1535, 1443, 1366, 1311, 1234, 1142, 1103, 1011. ^1^H NMR (DMSO-d_6_, 400 MHz, δ): 8.828 (s, 1H, Pyrazolyl H), 8.069-8.029 (m, 2H, Ar H), 7.745 (s, 1H, =CH), 7.685-7.649 (m, 2H, Ar H), 7.601-7.537 (m, 5H, Ar H), 7.453-7.417 (m, 1H, Ar H), 4.501 (s, 2H, NCH_2_), 3.711 (s, 3H, COOCH_3_). MS (ESI+) *m/z *406 [M+H]. Anal. Found: C, 62.7; H, 4.2; N, 9.9. C_22_H_17_N_3_O_4_S requires C, 63.00; H, 4.09; N, 10.02%.

### 4.13. Methyl 2-((Z)-2, 4-dioxo-5-((1-phenyl-3-p-tolyl-1H-pyrazol-4-yl)methylene)thiazolidin-3-yl)acetate (5b)

IR (ν_max_, KBr) cm^-1^: 1744, 1690, 1605, 1512, 1443, 1366, 1319, 1234, 1142, 1103, 1011. ^1^H NMR (TFA-d_1_, 400 MHz, δ): 8.501 (s, 1H, Pyrazolyl H), 7.924 (s, 1H, =CH), 7.626 (m, 5H, Ar H), 7.492-7.472 (m, 2H, Ar H), 7.417-7.398 (m, 2H, Ar H), 4.632 (s, 2H, NCH_2_), 3.711 (s, 3H, COOCH_3_), 2.404 (s, 3H, Ph CH_3_). MS (ESI+) *m/z *419 [M+H]. Anal. Found: C, 63.6; H, 4.5; N, 9.4. C_23_H_19_N_3_O_4_S requires C, 63.73; H, 4.42; N, 9.69%.

### 4.14. Methyl 2-((Z)-5-((3-(4-methoxyphenyl)-1-phenyl-1H-pyrazol-4-yl)methylene)-2, 4-dioxothiazolidin-3-yl)acetate (5c)

IR (ν_max_, KBr) cm^-1^: 1744, 1690, 1612, 1520, 1443, 1366, 1296, 1242, 1180, 1142, 1103, 1018. ^1^H NMR (TFA-d_1_, 400 MHz, δ): 8.477 (s, 1H, Pyrazolyl H), 7.915 (s, 1H, =CH), 7.665-7.568 (m, 6H, Ar H), 7.178-7.156 (d, 2H, Ar H, *J *= 8.8 Hz), 4.630 (s, 2H, NCH_2_), 3.923 (s, 3H, COOCH_3_), 3.859 (s, 3H, Ph OCH_3_). MS (ESI+) *m/z *436 [M+H]. Anal. Found: C, 61.3; H, 4.4; N, 9.2. C_23_H_19_N_3_O_5_S requires C, 61.46; H, 4.26; N, 9.35%.

### 4.15. Methyl 2-((Z)-5-((3-(4-chlorophenyl)-1-phenyl-1H-pyrazol-4-yl)methylene)-2, 4-dioxothiazolidin-3-yl)acetate (5d)

IR (ν_max_, KBr) cm^-1^: 1744, 1697, 1605, 1528, 1443, 1366, 1319, 1242, 1142, 1103, 1011. ^1^H NMR (TFA-d_1_, 400 MHz, δ): 8.476 (s, 1H, Pyrazolyl H), 7.884 (s, 1H, =CH), 7.618-7.552 (m, 9H, Ar H), 4.630 (s, 2H, NCH_2_), 3.861 (s, 3H, COOCH_3_). MS (ESI+) *m/z *440 [M+H]. Anal. Found: C, 58.0; H, 3.6; N, 9.1. C_22_H_16_N_3_O_4_S requires C, 58.21; H, 3.55; N, 9.26%.

### Methyl 2-((Z)-5-((3-(4-fluorophenyl)-1-phenyl-1H-pyrazol-4-yl)methylene)-2, 4-dioxothiazolidin-3-yl)acetate (5e)

IR (ν_max_, KBr) cm^-1^: 1744, 1697, 1612, 1520, 1404, 1366, 1319, 1234, 1149, 1095. ^1^H NMR (TFA-d_1_, 400 MHz, δ): 8.494 (s, 1H, Pyrazolyl H), 7.893 (s, 1H, =CH), 7.650-7.616 (m, 7H, Ar H), 7.300-7.258 (m, 2H, Ar H), 4.663 (s, 2H, NCH_2_), 3.876 (s, 3H, COOCH_3_). MS (ESI+) *m/z *424 [M+H]. Anal. Found: C, 60.2; H, 3.8; N, 9.5. C_22_H_16_FN_3_O_4_S requires C, 60.40; H, 3.69; N, 9.61%.

### Methyl 2-((Z)-5-((3-(4-bromophenyl)-1-phenyl-1H-pyrazol-4-yl)methylene)-2, 4-dioxothiazolidin-3-yl)acetate (5f)

IR (ν_max_, KBr) cm^-1^: 1744, 1697, 1612, 1520, 1404, 1366, 1319, 1234, 1149, 1095. ^1^H NMR (CDCl_3 _+ TFA-d_1_, 400 MHz, δ): 8.250 (s, 1H, Pyrazolyl H), 7.899 (s, 1H, =CH), 7.750-7.730 (d, 2H, Ar H, *J *= 8.0 Hz), 7.660-7.611 (m, 5H, Ar H), 7.500-7.480 (d, 2H, Ar H, *J *= 8.00 Hz), 4.652 (s, 2H, NCH_2_), 3.901 (s, 3H, COOCH_3_). MS (ESI+) *m/z *483 [M+H]. Anal. Found: C, 52.9; H, 3.4; N, 8.2. C_22_H_16_BrN_3_O_4_S requires C, 53.02; H, 3.24; N, 8.43%.

### Methyl 2-((Z)-5-((3-(4-hydroxyphenyl)-1-phenyl-1H-pyrazol-4-yl)methylene)-2, 4-dioxothiazolidin-3-yl)acetate (5g)

IR (ν_max_, KBr) cm^-1^: 3348, 1736, 1682, 1605, 1512, 1443, 1412, 1373, 1311, 1234, 1211, 1142, 1103. ^1^H NMR (DMSO-d_6_, 400 MHz, δ): 9.863 (s, 1H, Ph OH), 8.764 (s, 1H, Pyrazolyl H), 8.023-8.003 (m, 2H, Ar H), 7.730 (s, 1H, =CH), 7.585-7.546 (m, 2H, Ar H), 7.471-7.450 (d, 2H, Ar H, *J *= 8.4 Hz), 7.434-7.395 (m, 1H, Ar H), 6.954-6.933 (d, 2H, Ar H, *J *= 8.4 Hz), 4.499 (s, 2H, NCH_2_), 3.712 (s, 3H, COOCH_3_). MS (ESI+) *m/z *450 [M+H]. Anal. Found: C, 60.5; H, 4.0; N, 9.5. C_22_H_17_N_3_O_5_S requires C, 60.68; H, 3.93; N, 9.65%.

### Methyl 2-((Z)-5-((3-(4-nitrophenyl)-1-phenyl-1H-pyrazol-4-yl)methylene)-2, 4-dioxothiazolidin-3-yl)acetate (5h)

IR (ν_max_, KBr) cm^-1^: 1744, 1690, 1605, 1528, 1412, 1342, 1273, 1219, 1142, 1103. ^1^H NMR (CDCl_3 _+ TFA-d_1_, 400 MHz, δ): 8.454-8.434 (d, 2H, Ar H, *J *= 8.8 Hz), 8.261-8.247 (m, 2H, Ar H), 7.906-7.834 (m, 3H, Ar H), 7.710-7.689 (m, 2H, Ar H), 7.637-7.571 (m, 2H, Ar H), 4.642 (s, 2H, NCH_2_), 3.985 (s, 3H, COOCH_3_). MS (ESI+) *m/z *450 [M+H]. Anal. Found: C, 58.7; H, 3.6; N, 11.8. C_22_H_16_N_4_O_6_S requires C, 58.89; H, 3.47; N, 12.06%.

### Preparation of 2-((*Z*)-5-((3-aryl-1-phenyl-1*H*-pyrazol-4-yl)methylene)-2, 4-dioxothiazolidin-3-yl)acetic acid (6a-h)

Typical procedure: A mixture of ethyl 2-((*Z*)-2, 4-dioxo-5-((1, 3-diphenyl-1*H*-pyrazol-4-yl)methylene)thiazolidin-3-yl)acetate **4a **(0.5g, 1.1 mmol), 10 mL of 50% aqueous sulphuric acid in 35 mL acetic acid was refluxed for 5-6 h. On cooling, the reaction mixture was poured onto crushed ice. Solid separated was filtered, washed with excess of cold water followed by alcohol to obtain white solid **6a **(0.47g, 94%). Similarly, **6a **can also be obtained from **5a **by hydrolysis.

All other derivatives **6b-h **were synthesized by adopting the similar procedure.

### 2-((Z)-2, 4-Dioxo-5-((1, 3-diphenyl-1H-pyrazol-4-yl)methylene)thiazolidin-3-yl)acetic acid (6a)

IR (ν_max_, KBr) cm^-1^: 3472, 3418, 1744, 1697, 1605, 1528, 1504, 1443, 1373, 1319, 1219, 1149, 1103, 1102, 1057, 1003. ^1^H NMR (DMSO-d_6_, 300 MHz, δ): 8.807 (s, 1H, Pyrazolyl H), 8.040-8.018 (m, 2H, Ar H), 7.729-7.434 (m, 9H, ArH + =CH), 4.359 (s, 2H, NCH_2_). MS (ESI+) *m/z *392 [M+H]. Anal. Found: C, 62.1; H, 3.8; N, 10.2. C_21_H_15_N_3_O_4_S requires C, 62.21; H, 3.73; N, 10.36%.

### 2-((Z)-2, 4-Dioxo-5-((1-phenyl-3-p-tolyl-1H-pyrazol-4-yl)methylene)thiazolidin-3-yl)acetic acid (6b)

IR (ν_max_, KBr) cm^-1^: 1744, 1697, 1605, 1512, 1450, 1389, 1319, 1227, 1149, 1103, 1003. ^1^H NMR (DMSO-d_6_, 300 MHz, δ): 8.795 (s, 1H, Pyrazolyl H), 8.045-8.015 (m, 2H, Ar H), 7.727 (s, 1H, =CH), 7.603-7.530 (m, 4H, Ar H), 7.451-7.373 (m, 3H, Ar H), 4.366 (s, 2H, NCH_2_), 2.405 (s, 3H, CH_3_). MS (ESI+) *m/z *406 [M+H]. Anal. Found: C, 62.8; H, 4.2; N, 9.9. C_22_H_17_N_3_O_4_S requires C, 63.00; H, 4.09; N, 10.02%.

### 2-((Z)-5-((3-(4-Methoxyphenyl)-1-phenyl-1H-pyrazol-4-yl)methylene)-2, 4-dioxothiazolidin-3-yl)acetic acid (6c)

IR (ν_max_, KBr) cm^-1^: 1736, 1690, 1612, 1520, 1450, 1396, 1296, 1242, 1180, 1142, 1103, 1018. ^1^H NMR (DMSO-d_6_, 300 MHz, δ): 8.782 (s, 1H, Pyrazolyl H), 8.037-8.011 (m, 2H, Ar H), 7.722 (s, 1H, =CH), 7.599-7.548 (m, 4H, Ar H), 7.447-7.398 (m, 1H, Ar H), 7.149-7.120 (d, 2H, Ar H, *J *= 8.7 Hz), 4.365 (s, 2H, NCH_2_), 3.842 (s, 3H, OCH_3_). MS (ESI+) *m/z *422 [M+H]. Anal. Found: C, 60.5; H, 3.8, N, 14.20. C_22_H_17_N_3_O_5_S requires C, 60.68; H, 3.93; N, 9.65%.

### 2-((Z)-5-((3-(4-Chlorophenyl)-1-phenyl-1H-pyrazol-4-yl)methylene)-2, 4-dioxothiazolidin-3-yl)acetic acid (6d)

IR (ν_max_, KBr) cm^-1^: 3472, 3418, 1736, 1690, 1612, 1520, 1450, 1396, 1296, 1242, 1180, 1142, 1103, 1018. ^1^H NMR (DMSO-d_6_, 300 MHz, δ): 8.776 (s, 1H, Pyrazolyl H), 8.006-7.980 (d, 2H, Ar H, *J *= 7.8 Hz), 7.687 (s, 1H, =CH), 7.656-7.544 (m, 6H, Ar H), 7.449-7.365 (m, 1H, Ar H), 4.350 (s, 2H, NCH_2_). MS (ESI+) *m/z *426 [M+H]. Anal. Found: C, 57.0; H, 3.4; N, 9.4. C_21_H_14_ClN_3_O_4_S requires C, 57.34; H, 3.21; N, 9.55%.

### 2-((Z)-5-((3-(4-Fluorophenyl)-1-phenyl-1H-pyrazol-4-yl)methylene)-2, 4-dioxothiazolidin-3-yl)acetic acid (6e)

IR (ν_max_, KBr) cm^-1^: 1751, 1697, 1612, 1512, 1450, 1373, 1319, 1227, 1149, 1095, 1003. ^1^H NMR (DMSO-d_6_, 300 MHz, δ): 8.819 (s, 1H, Pyrazolyl H), 8.048-8.022 (d, 2H, Ar H, *J *= 7.8 Hz), 7.737-7.711 (m, 3H, =CH and Ar H), 7.607-7.556 (m, 2H, Ar H), 7.455-7.396 (m, 3H, Ar H), 4.369 (s, 2H, NCH_2_). MS (ESI+) *m/z *410 [M+H]. Anal. Found: C, 59.4; H, 3.5; N, 9.8. C_21_H_14_FN_3_O_4_S requires C, 59.57; H, 3.33; N, 9.92%.

### 2-((Z)-5-((3-(4-Bromophenyl)-1-phenyl-1H-pyrazol-4-yl)methylene)-2, 4-dioxothiazolidin-3-yl)acetic acid (6f)

IR (ν_max_, KBr) cm^-1^: 1744, 1697, 1605, 1528, 1504, 1443, 1389, 1319, 1242, 1149, 1103, 1003. ^1^H NMR (DMSO-d_6_, 300 MHz, δ): 8.822 (s, 1H, Pyrazolyl H), 8.039-8.013 (m, 2H, Ar H), 7.798-7.771 (d, 2H, Ar H, *J *= 8.1 Hz), 7.712 (s, 1H, =CH), 7.634-7.607 (d, 2H, Ar H, *J *= 8.1 Hz), 7.581-7.555 (m, 2H, Ar H), 7.460-7.413 (m, 1H, Ar H), 4.372 (s, 2H, NCH_2_). MS (ESI+) *m/z *470 [M+H]. Anal. Found: C, 51.9; H, 2.8; N, 8.5. C_21_H_14_BrN_3_O_4_S requires C, 52.08; H, 2.91; N, 8.68%.

### 2-((Z)-5-((3-(4-Hydroxyphenyl)-1-phenyl-1H-pyrazol-4-yl)methylene)-2, 4-dioxothiazolidin-3-yl)acetic acid (6g)

IR (ν_max_, KBr) cm^-1^: 3379, 3310, 1736, 1713, 1674, 1605, 1512, 1443, 1404, 1373, 1219, 1142, 1103, 1057, 1003. ^1^H NMR (DMSO-d_6_, 300 MHz, δ): 9.886 (bs, 1H, Ph OH), 8.753 (s, 1H, Pyrazolyl H), 8.026-8.000 (d, 2H, Ar H, *J *= 7.8 Hz), 7.721 (s, 1H, =CH), 7.591-7.540 (m, 2H, Ar H), 7.476-7.388 (m, 3H, Ar H), 6.960-6.933 (d, 2H, Ar H, *J *= 8.1 Hz), 4.361 (s, 2H, NCH_2_). MS (ESI+) *m/z *408 [M+H]. Anal. Found: C, 59.7; H, 3.7; N, 9.8. C_21_H_15_N_3_O_5_S requires C, 59.85; H, 3.59; N, 9.97%.

### 2-((Z)-5-((3-(4-Nitrophenyl)-1-phenyl-1H-pyrazol-4-yl)methylene)-2, 4-dioxothiazolidin-3-yl)acetic acid (6h)

IR (ν_max_, KBr) cm^-1^: 3418, 3479, 1774, 1728, 1674, 1605, 1528, 1404, 1350, 1242, 1180, 1142, 1103 1065. ^1^H NMR (DMSO-d_6_, 300 MHz, δ): 8.887 (s, 1H, Pyrazolyl H), 8.433-8.404 (d, 2H, Ar H, *J *= 8.7 Hz), 8.066-8.039 (d, 2H, Ar H, J = 8.1 Hz), 7.983-7.954 (d, 2H, Ar H, *J *= 8.7 Hz), 7.763 (s, 1H, =CH), 7.622-7.571 (m, 2H, Ar H), 7.482-7.434 (m, 1H, Ar H), 4.384 (s, 2H, NCH_2_). MS (ESI+) *m/z *451 [M+H]. Anal. Found: C, 55.8; H, 3.0; N, 12.3. C_21_H_14_N_4_O_6_S requires C, 56.00; H, 3.13; N, 12.44%.

## Biological assay

### Test microorganisms

Four bacteria, *S. aureus *(MTCC 96), *B. subtilis *(MTCC 121) (Gram-positive), *E. coli *(MTCC 1652) and *P. aeruginosa *(MTCC 741) (Gram-negative) procured from MTCC, Chandigarh and two fungi, *A. niger *and *A. flavus*, the ear pathogens isolated from the Kurukshetra patients, were used in this study [52].

### *In vitro *antibacterial activity

The antibacterial activity of synthesized compounds was evaluated by the agar well-diffusion method. All the cultures were adjusted to 0.5 McFarland standard, which is visually comparable to a microbial suspension of approximately 1.5 × 10^8 ^cfu/mL. 20-mL of Mueller Hinton agar medium was poured into each Petri plate and the agar plates were swabbed with 100 μL inocula of each test bacterium and kept for 15 min for adsorption. Using sterile cork borer of 8-mm diameter, wells were bored into the seeded agar plates and these were loaded with a 100-μL volume with concentration of 4.0 mg/mL of each compound reconstituted in the dimethylsulphoxide (DMSO). All the plates were incubated at 37°C for 24 h. Antibacterial activity of each synthetic compound was evaluated by measuring the zone of growth inhibition against the test organisms with zone reader (Hi Antibiotic zone scale). DMSO was used as a negative control whereas ciprofloxacin was used as a positive control. This procedure was performed in three replicate plates for each organism [53].

### Determination of MIC

MIC is the lowest concentration of an antimicrobial compound that will inhibit the visible growth of a microorganism after overnight incubation. MIC of the various compounds against bacterial strains was tested through a macro dilution tube method as recommended by NCCLS [54]. In this method, various test concentrations of synthesized compounds were made from 128 to 0.25 μg/mL in sterile tubes no. 1 to 10. 100-μL sterile Mueller Hinton Broth (MHB) was poured in each sterile tube followed by addition of 200 μL test compound in tube 1. Twofold serial dilutions were carried out from the tube no. 1 to the tube no. 10 and excess broth (100 μL) was discarded from the last tube no. 10. To each tube, 100 μL of standard inoculums (1.5 × 10^8 ^cfu/mL) was added. Ciprofloxacin was used as control. Turbidity was observed after incubating the inoculated tubes at 37°C for 24 h.

### *In vitro *antifungal activity

The antifungal activity of the synthesized compounds was evaluated by poisoned food technique. The molds were grown on Sabouraud dextrose agar (SDA) at 25°C for 7 days and used as inocula. 15 mL of molten SDA (45°C) was poisoned by the addition of 100 μL volume of each compound having concentration of 4.0 mg/mL, reconstituted in the DMSO, poured into a sterile Petri plate and allowed it to solidify at room temperature. The solidified poisoned agar plates were inoculated at the centre with fungal plugs (8-mm diameter), obtained from the actively growing colony and incubated at 25°C for 7 days. DMSO was used as the negative control whereas fluconazole was used as the positive control. The experiments were performed in triplicates. Diameter of the fungal colonies was measured and expressed as percent mycelial inhibition determined by applying the formula [55].

Inhibition of mycelial growth %= (dc-dt)∕dc×100

where *dc *average diameter of fungal colony in negative control plates, *dt *average diameter of fungal colony in experimental plates.

## Abbreviations

DMSO: dimethylsulfoxide; MIC: minimum inhibitory concentration; MTCC: microbial-type culture collection; SDA: Sabouraud dextrose agar; TZDs: thiazolidine-2,4-dione.

## Competing interests

The authors declare that they have no competing interests.

## Supplementary Material

Additional file 1 **^1^H NMR Spectra **.(4a); ^1^H NMR of ethyl 2-((Z)-2, 4-dioxo-5-((1, 3-diphenyl-1H-pyrazol-4-yl)methylene)thiazolidin-3-yl)acetate Click here for file

Additional file 2 **^1^H NMR Spectra **.(4b); ^1^H NMR of ethyl 2-((Z)-2, 4-dioxo-5-((1-phenyl-3-p-tolyl-1H-pyrazol-4-yl)methylene)thiazolidin-3-yl)acetate Click here for file

Additional file 3 **^1^H NMR Spectra **.(4c); ^1^H NMR of ethyl 2-((Z)-5-((3-(4-methoxyphenyl)-1-phenyl-1H-pyrazol-4-yl)methylene)-2, 4-dioxothiazolidin-3-yl)acetate Click here for file

Additional file 4 **^1^H NMR Spectra **.(4d); ^1^H NMR of ethyl 2-((Z)-5-((3-(4-chlorophenyl)-1-phenyl-1H-pyrazol-4-yl)methylene)-2, 4-dioxothiazolidin-3-yl)acetate Click here for file

Additional file 5 **^1^H NMR Spectra **.(4e); ^1^H NMR of ethyl 2-((Z)-5-((3-(4-fluorophenyl)-1-phenyl-1H-pyrazol-4-yl)methylene)-2, 4-dioxothiazolidin-3-yl)acetate Click here for file

Additional file 6 **^1^H NMR Spectra **.(4f); ^1^H NMR of ethyl 2-((Z)-5-((3-(4-bromophenyl)-1-phenyl-1H-pyrazol-4-yl)methylene)-2, 4-dioxothiazolidin-3-yl)acetate Click here for file

Additional file 7 **^1^H NMR Spectra **.(4g); ^1^H NMR of ethyl 2-((Z)-5-((3-(4-hydroxyphenyl)-1-phenyl-1H-pyrazol-4-yl)methylene)-2, 4-dioxothiazolidin-3-yl)acetate Click here for file

Additional file 8 **^1^H NMR Spectra **.(4h); ^1^H NMR of ethyl 2-((Z)-5-((3-(4-nitrophenyl)-1-phenyl-1H-pyrazol-4-yl)methylene)-2, 4-dioxothiazolidin-3-yl)acetate Click here for file

Additional file 9 **^1^H NMR Spectra **.(5a); ^1^H NMR of methyl 2-((Z)-2, 4-dioxo-5-((1, 3-diphenyl-1H-pyrazol-4-yl)methylene)thiazolidin-3-yl)acetate Click here for file

Additional file 10 **^1^H NMR Spectra **.(5b); ^1^H NMR of methyl 2-((Z)-2, 4-dioxo-5-((1-phenyl-3-p-tolyl-1H-pyrazol-4-yl)methylene)thiazolidin-3-yl)acetate Click here for file

Additional file 11 **^1^H NMR Spectra **.(5c); ^1^H NMR of methyl 2-((Z)-5-((3-(4-methoxyphenyl)-1-phenyl-1H-pyrazol-4-yl)methylene)-2, 4-dioxothiazolidin-3-yl)acetate Click here for file

Additional file 12 **^1^H NMR Spectra **.(5d); ^1^H NMR of methyl 2-((Z)-5-((3-(4-chlorophenyl)-1-phenyl-1H-pyrazol-4-yl)methylene)-2, 4-dioxothiazolidin-3-yl)acetate Click here for file

Additional file 13 **^1^H NMR Spectra **.(5e); ^1^H NMR of methyl 2-((Z)-5-((3-(4-fluorophenyl)-1-phenyl-1H-pyrazol-4-yl)methylene)-2, 4-dioxothiazolidin-3-yl)acetate Click here for file

Additional file 14 **^1^H NMR Spectra **.(5f); ^1^H NMR of methyl 2-((Z)-5-((3-(4-bromophenyl)-1-phenyl-1H-pyrazol-4-yl)methylene)-2, 4-dioxothiazolidin-3-yl)acetate Click here for file

Additional file 15 **^1^H NMR Spectra **.(5g); ^1^H NMR of methyl 2-((Z)-5-((3-(4-hydroxyphenyl)-1-phenyl-1H-pyrazol-4-yl)methylene)-2, 4-dioxothiazolidin-3-yl)acetate Click here for file

Additional file 16 **^1^H NMR Spectra **.(5h); ^1^H NMR of methyl 2-((Z)-5-((3-(4-nitrophenyl)-1-phenyl-1H-pyrazol-4-yl)methylene)-2, 4-dioxothiazolidin-3-yl)acetate Click here for file

Additional file 17 **^1^H NMR Spectra **.(6a); ^1^H NMR of 2-((Z)-2, 4-dioxo-5-((1, 3-diphenyl-1H-pyrazol-4-yl)methylene)thiazolidin-3-yl)acetic acid Click here for file

Additional file 18 **^1^H NMR Spectra **.(6b); ^1^H NMR of 2-((Z)-2, 4-dioxo-5-((1-phenyl-3-p-tolyl-1H-pyrazol-4-yl)methylene)thiazolidin-3-yl)acetic acid Click here for file

Additional file 19 **^1^H NMR Spectra **.(6c); ^1^H NMR of 2-((Z)-5-((3-(4-methoxyphenyl)-1-phenyl-1H-pyrazol-4-yl)methylene)-2, 4-dioxothiazolidin-3-yl)acetic acid Click here for file

Additional file 20 **^1^H NMR Spectra **.(6d); ^1^H NMR of 2-((Z)-5-((3-(4-chlorophenyl)-1-phenyl-1H-pyrazol-4-yl)methylene)-2, 4-dioxothiazolidin-3-yl)acetic acid Click here for file

Additional file 21 **^1^H NMR Spectra **.(6e); ^1^H NMR of 2-((Z)-5-((3-(4-fluorophenyl)-1-phenyl-1H-pyrazol-4-yl)methylene)-2, 4-dioxothiazolidin-3-yl)acetic acid Click here for file

Additional file 22 **^1^H NMR Spectra **.(6f); ^1^H NMR of 2-((Z)-5-((3-(4-bromophenyl)-1-phenyl-1H-pyrazol-4-yl)methylene)-2, 4-dioxothiazolidin-3-yl)acetic acid Click here for file

Additional file 23 **^1^H NMR Spectra **.(6g); ^1^H NMR of 2-((Z)-5-((3-(4-hydroxyphenyl)-1-phenyl-1H-pyrazol-4-yl)methylene)-2, 4-dioxothiazolidin-3-yl)acetic acid Click here for file

Additional file 24 **^1^H NMR Spectra **.(6h); ^1^H NMR of 2-((Z)-5-((3-(4-nitrophenyl)-1-phenyl-1H-pyrazol-4-yl)methylene)-2, 4-dioxothiazolidin-3-yl)acetic acid Click here for file
