# Supplementary figures and images for: Synthesis of new pyrazolyl-2, 4-thiazolidinediones as antibacterial and antifungal agents
Source: Org Med Chem Lett. 2011 Nov 8;1:15. doi: 10.1186/2191-2858-1-15 (PMC3320062; doi:10.1186/2191-2858-1-15)

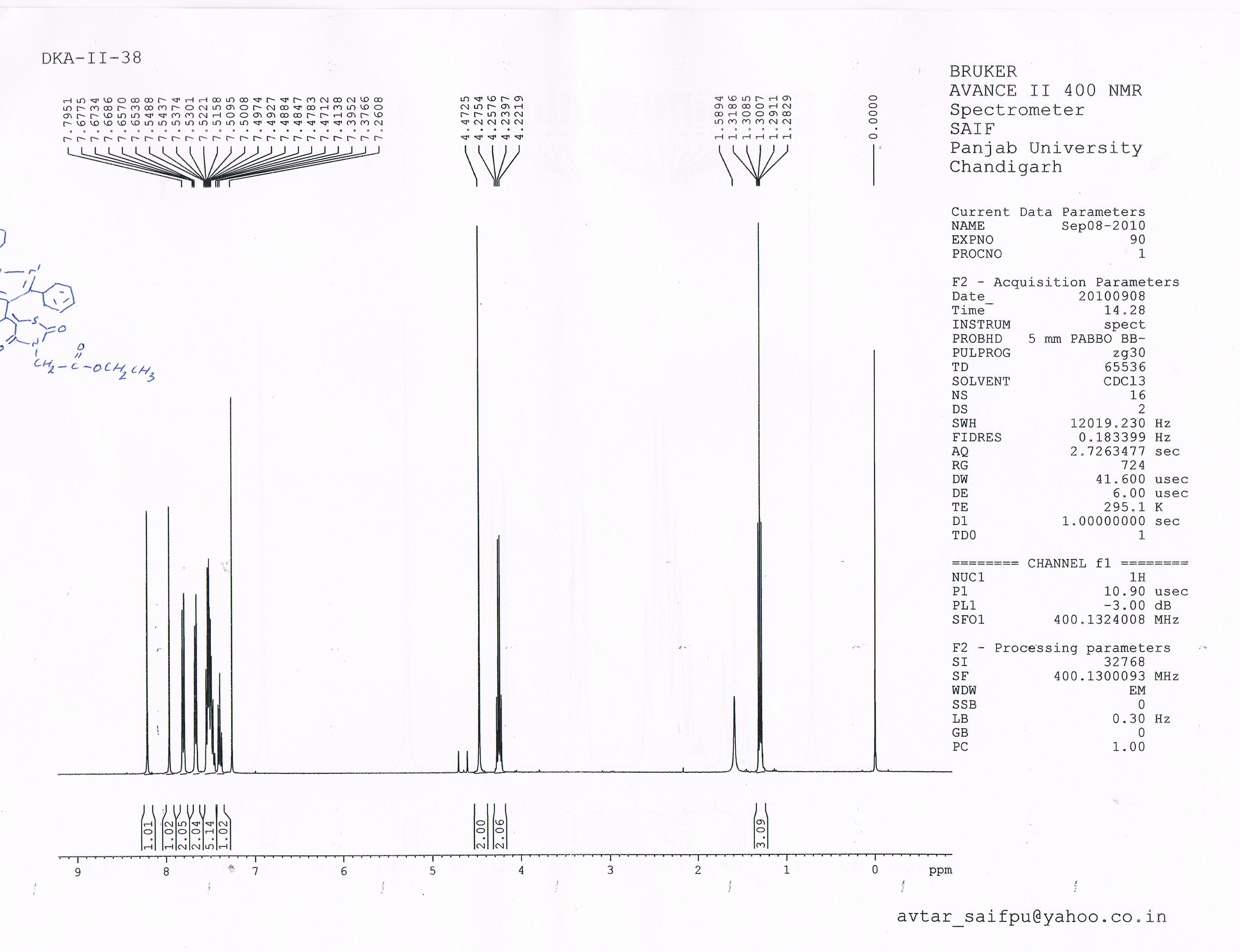

Supplement: Additional file 1 — 1H NMR Spectra .(4a); 1H NMR of ethyl 2-((Z)-2, 4-dioxo-5-((1, 3-diphenyl-1H-pyrazol-4-yl)methylene)thiazolidin-3-yl)acetate [file 2191-2858-1-15-S1.JPEG]

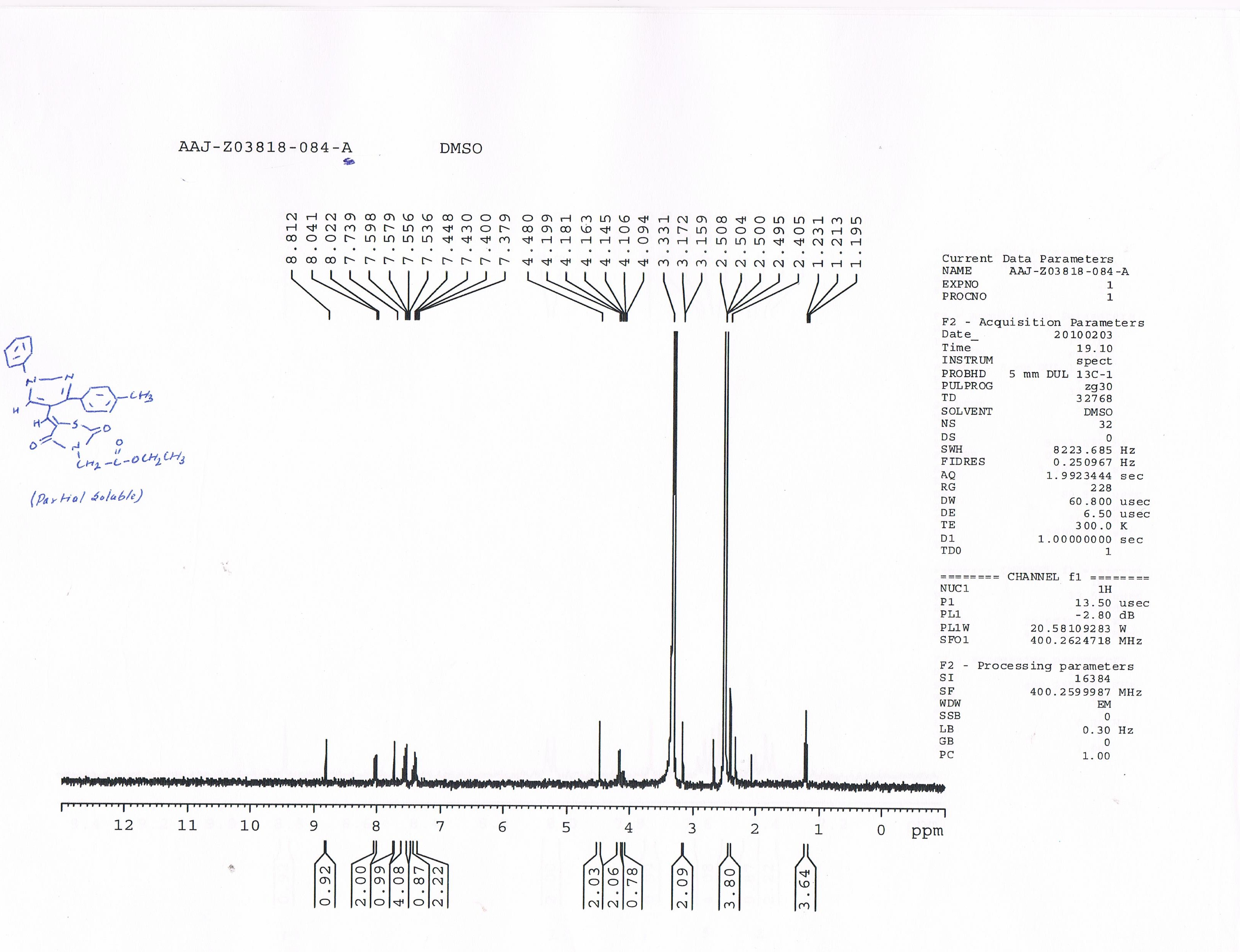

Supplement: Additional file 2 — 1H NMR Spectra .(4b); 1H NMR of ethyl 2-((Z)-2, 4-dioxo-5-((1-phenyl-3-p-tolyl-1H-pyrazol-4-yl)methylene)thiazolidin-3-yl)acetate [file 2191-2858-1-15-S2.JPEG]

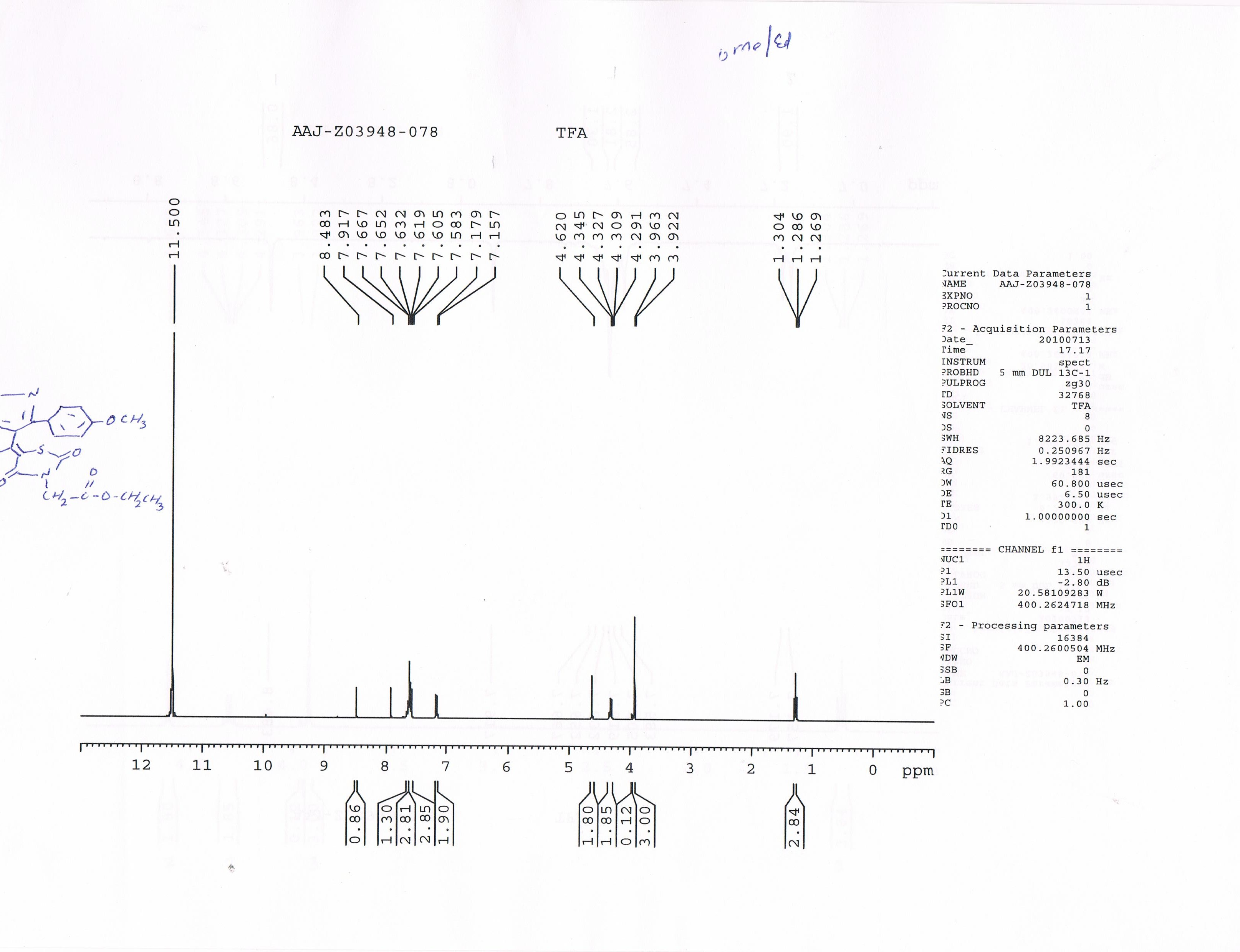

Supplement: Additional file 3 — 1H NMR Spectra .(4c); 1H NMR of ethyl 2-((Z)-5-((3-(4-methoxyphenyl)-1-phenyl-1H-pyrazol-4-yl)methylene)-2, 4-dioxothiazolidin-3-yl)acetate [file 2191-2858-1-15-S3.JPEG]

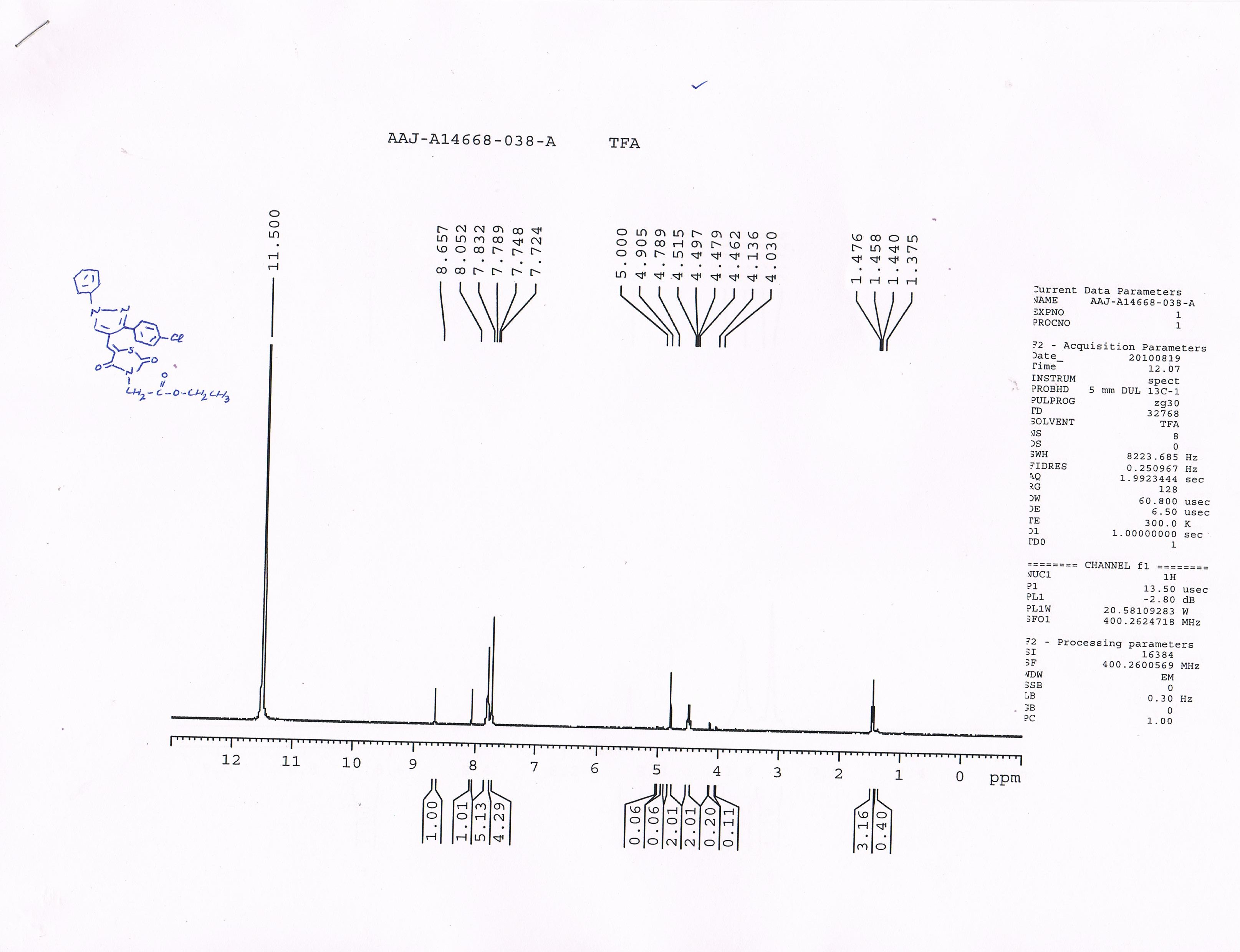

Supplement: Additional file 4 — 1H NMR Spectra .(4d); 1H NMR of ethyl 2-((Z)-5-((3-(4-chlorophenyl)-1-phenyl-1H-pyrazol-4-yl)methylene)-2, 4-dioxothiazolidin-3-yl)acetate [file 2191-2858-1-15-S4.JPEG]

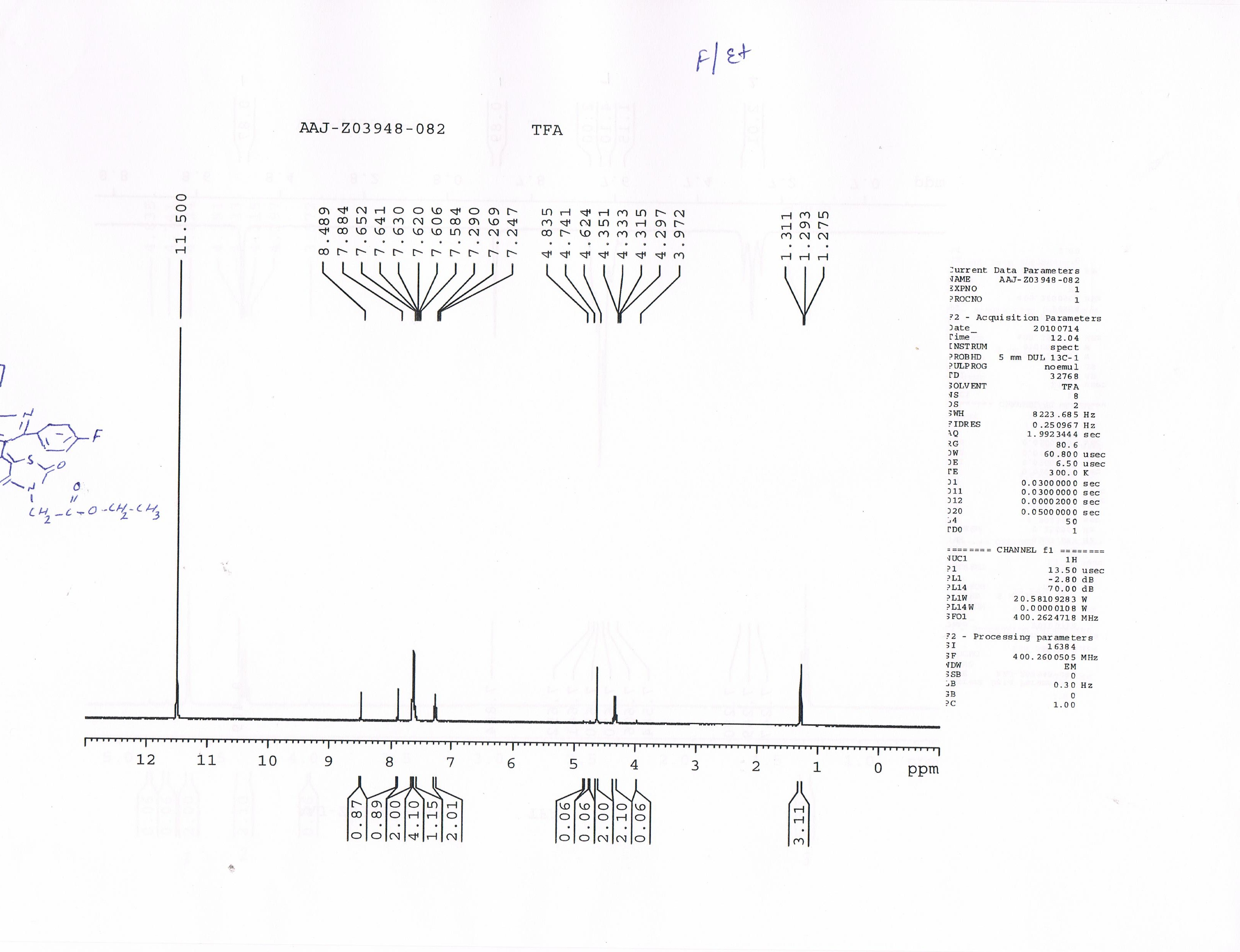

Supplement: Additional file 5 — 1H NMR Spectra .(4e); 1H NMR of ethyl 2-((Z)-5-((3-(4-fluorophenyl)-1-phenyl-1H-pyrazol-4-yl)methylene)-2, 4-dioxothiazolidin-3-yl)acetate [file 2191-2858-1-15-S5.JPEG]

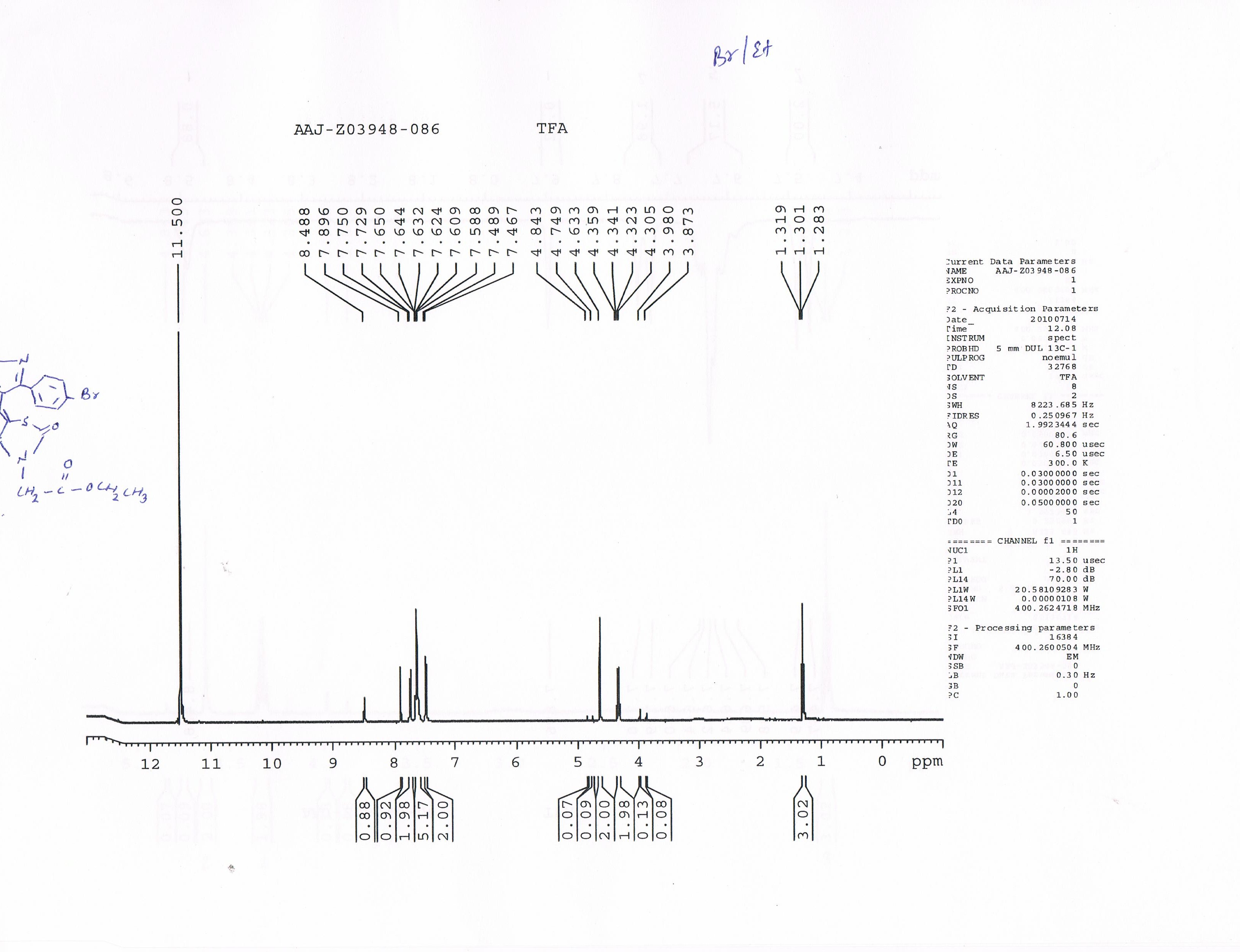

Supplement: Additional file 6 — 1H NMR Spectra .(4f); 1H NMR of ethyl 2-((Z)-5-((3-(4-bromophenyl)-1-phenyl-1H-pyrazol-4-yl)methylene)-2, 4-dioxothiazolidin-3-yl)acetate [file 2191-2858-1-15-S6.JPEG]

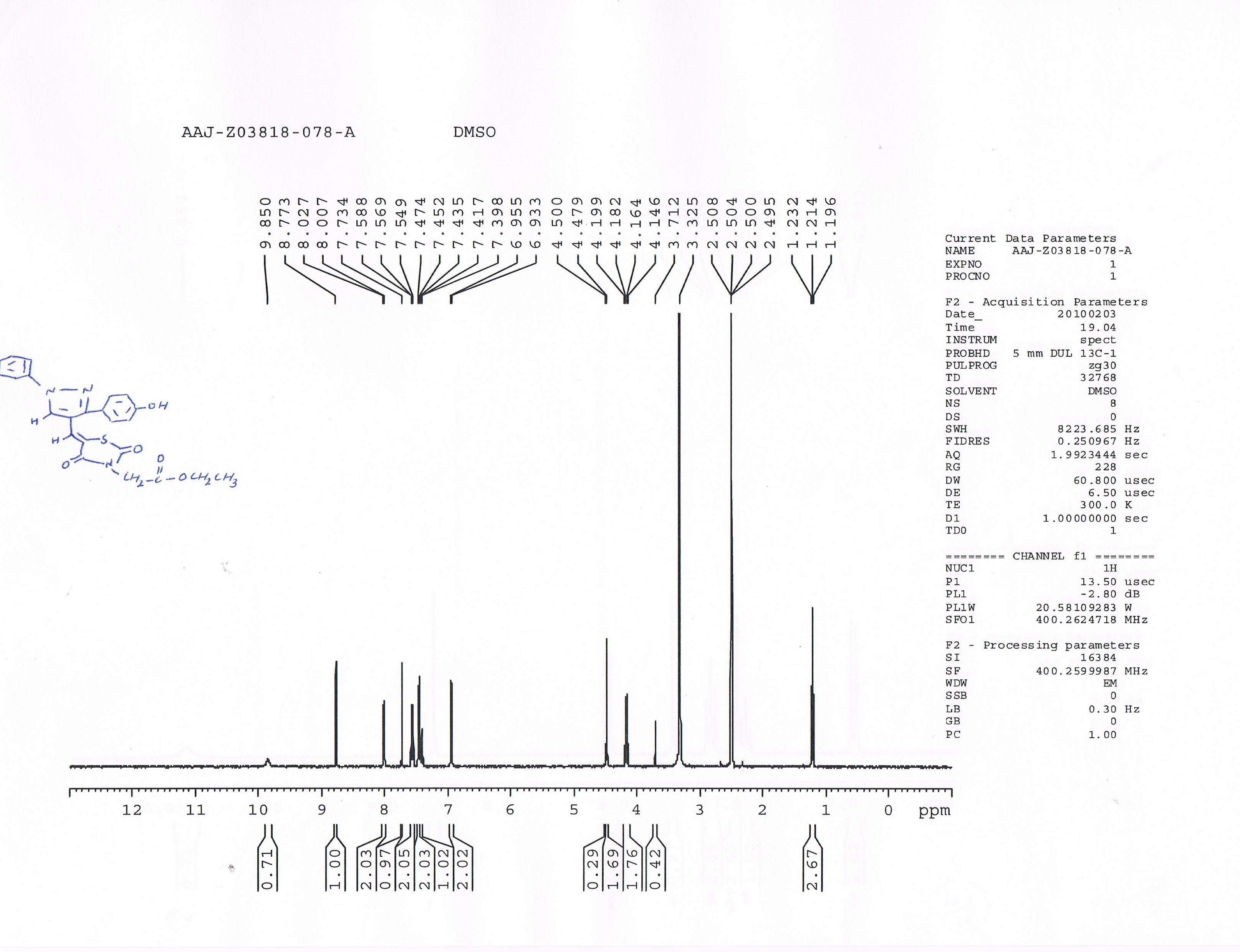

Supplement: Additional file 7 — 1H NMR Spectra .(4g); 1H NMR of ethyl 2-((Z)-5-((3-(4-hydroxyphenyl)-1-phenyl-1H-pyrazol-4-yl)methylene)-2, 4-dioxothiazolidin-3-yl)acetate [file 2191-2858-1-15-S7.JPEG]

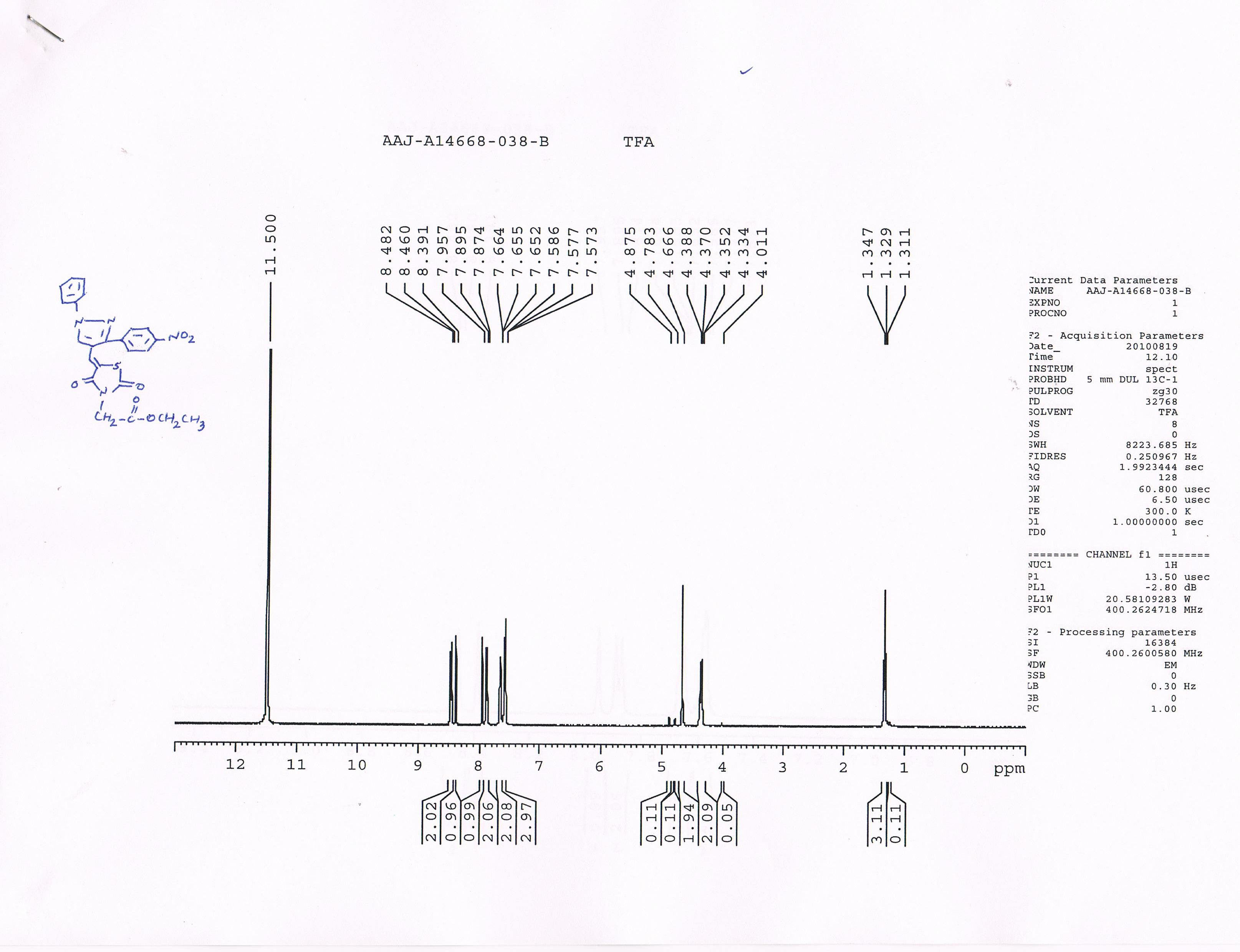

Supplement: Additional file 8 — 1H NMR Spectra .(4h); 1H NMR of ethyl 2-((Z)-5-((3-(4-nitrophenyl)-1-phenyl-1H-pyrazol-4-yl)methylene)-2, 4-dioxothiazolidin-3-yl)acetate [file 2191-2858-1-15-S8.JPEG]

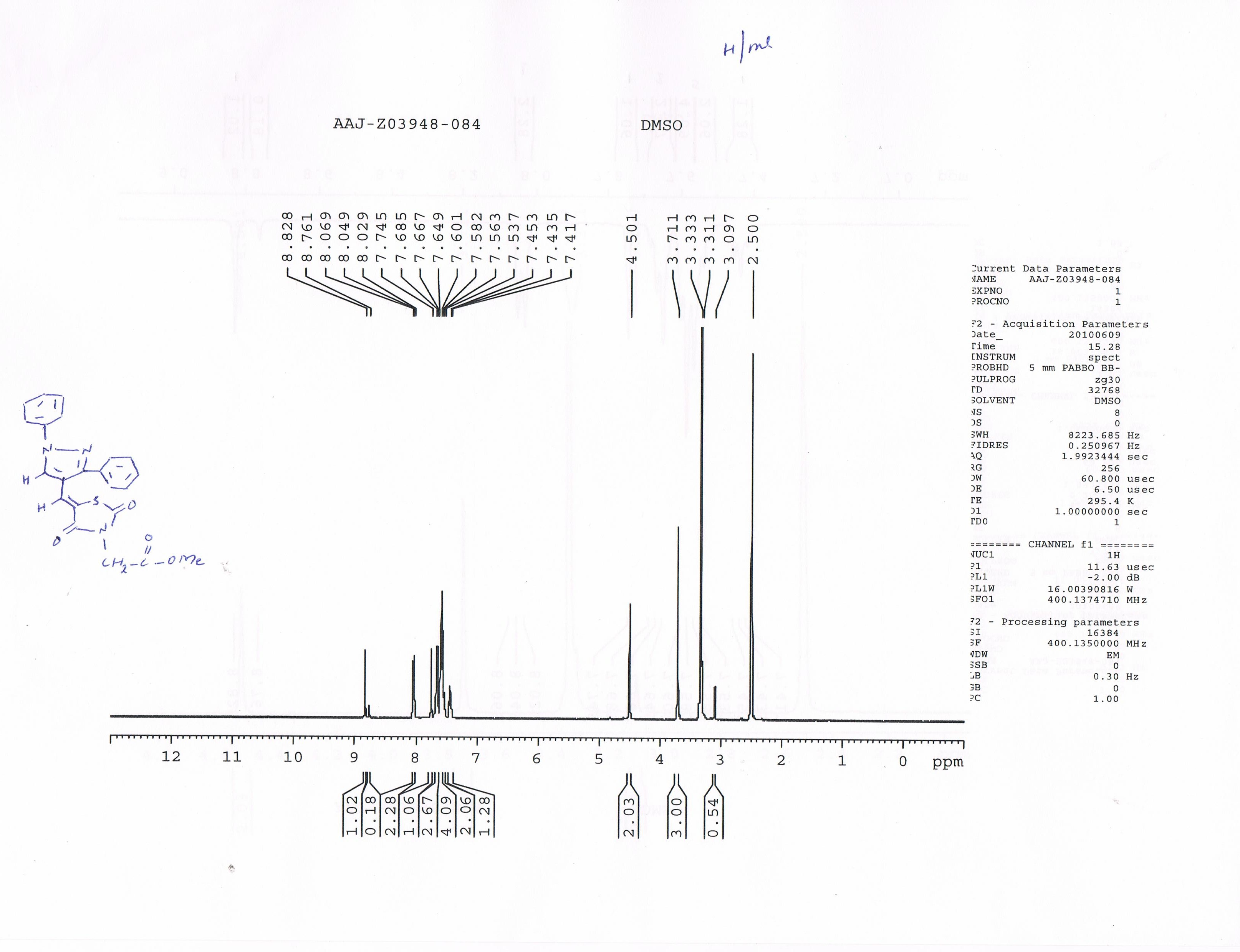

Supplement: Additional file 9 — 1H NMR Spectra .(5a); 1H NMR of methyl 2-((Z)-2, 4-dioxo-5-((1, 3-diphenyl-1H-pyrazol-4-yl)methylene)thiazolidin-3-yl)acetate [file 2191-2858-1-15-S9.JPEG]

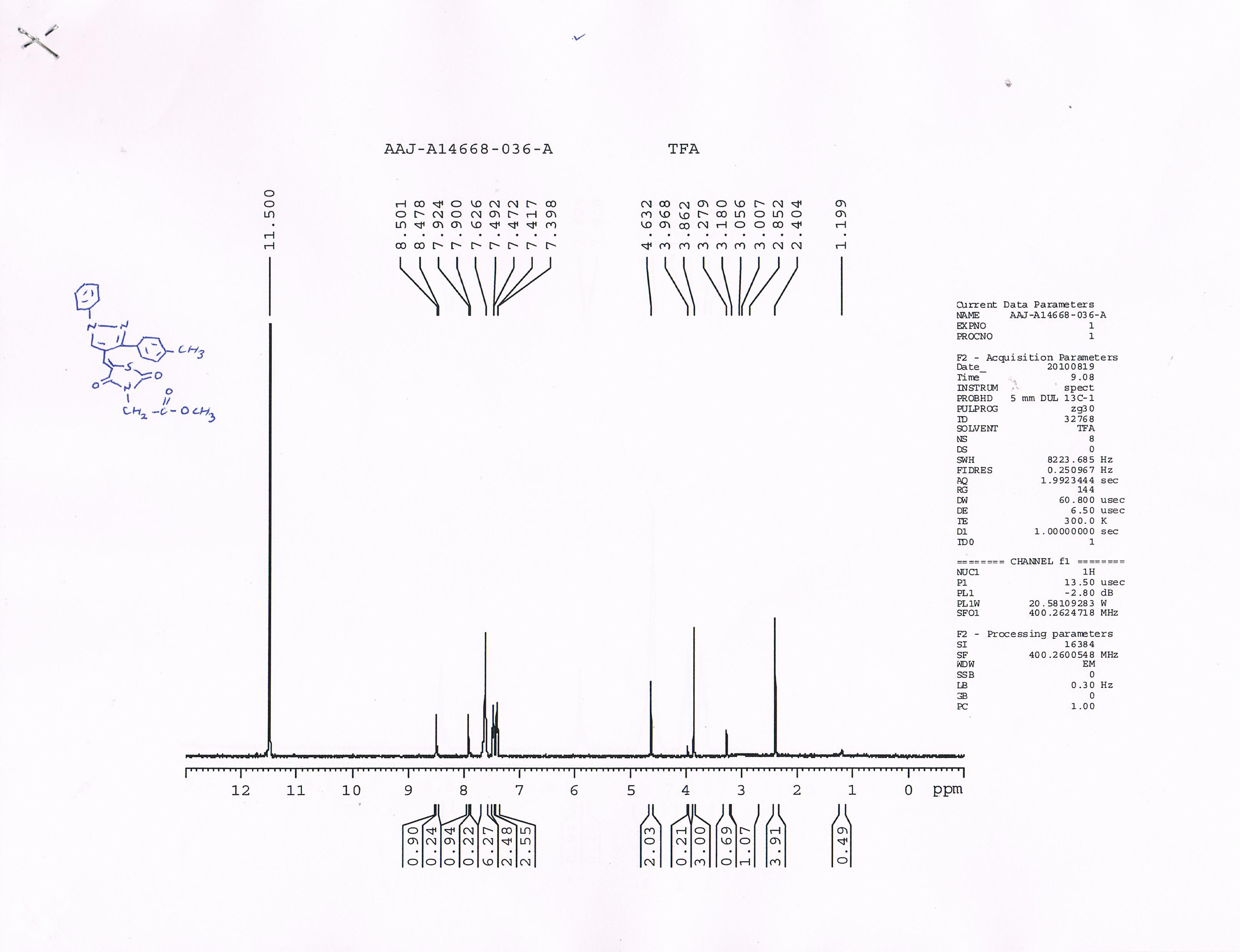

Supplement: Additional file 10 — 1H NMR Spectra .(5b); 1H NMR of methyl 2-((Z)-2, 4-dioxo-5-((1-phenyl-3-p-tolyl-1H-pyrazol-4-yl)methylene)thiazolidin-3-yl)acetate [file 2191-2858-1-15-S10.JPEG]

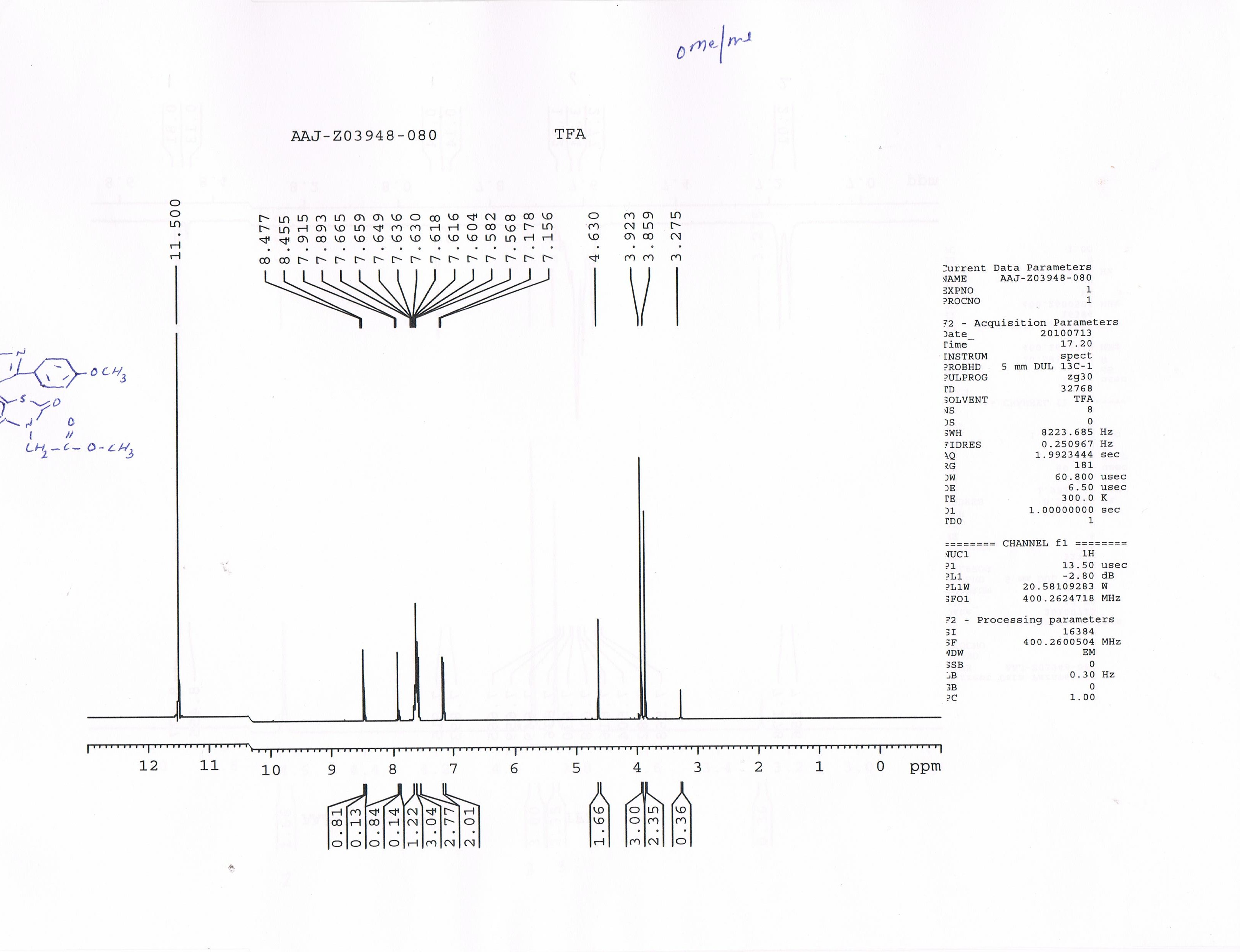

Supplement: Additional file 11 — 1H NMR Spectra .(5c); 1H NMR of methyl 2-((Z)-5-((3-(4-methoxyphenyl)-1-phenyl-1H-pyrazol-4-yl)methylene)-2, 4-dioxothiazolidin-3-yl)acetate [file 2191-2858-1-15-S11.JPEG]

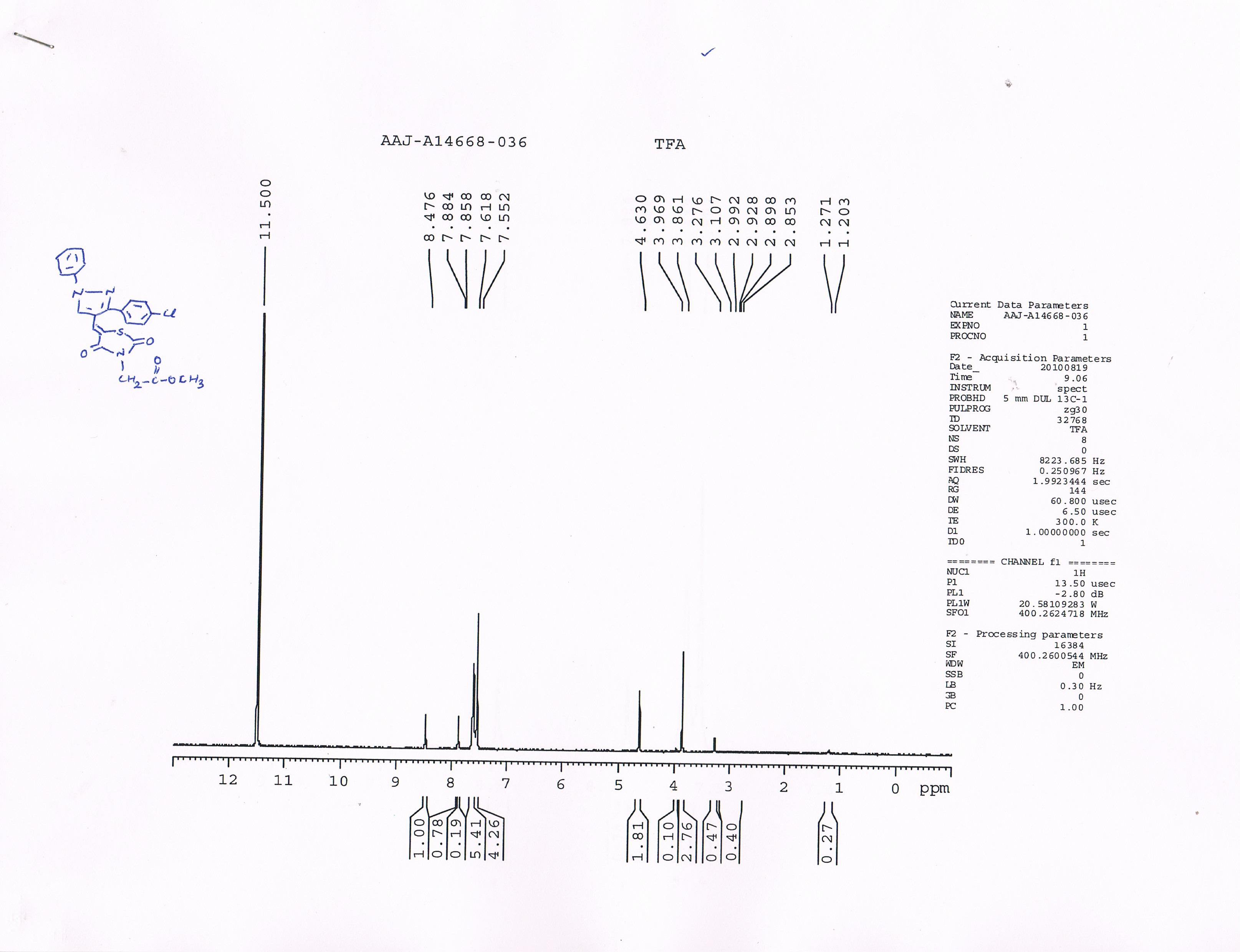

Supplement: Additional file 12 — 1H NMR Spectra .(5d); 1H NMR of methyl 2-((Z)-5-((3-(4-chlorophenyl)-1-phenyl-1H-pyrazol-4-yl)methylene)-2, 4-dioxothiazolidin-3-yl)acetate [file 2191-2858-1-15-S12.JPEG]

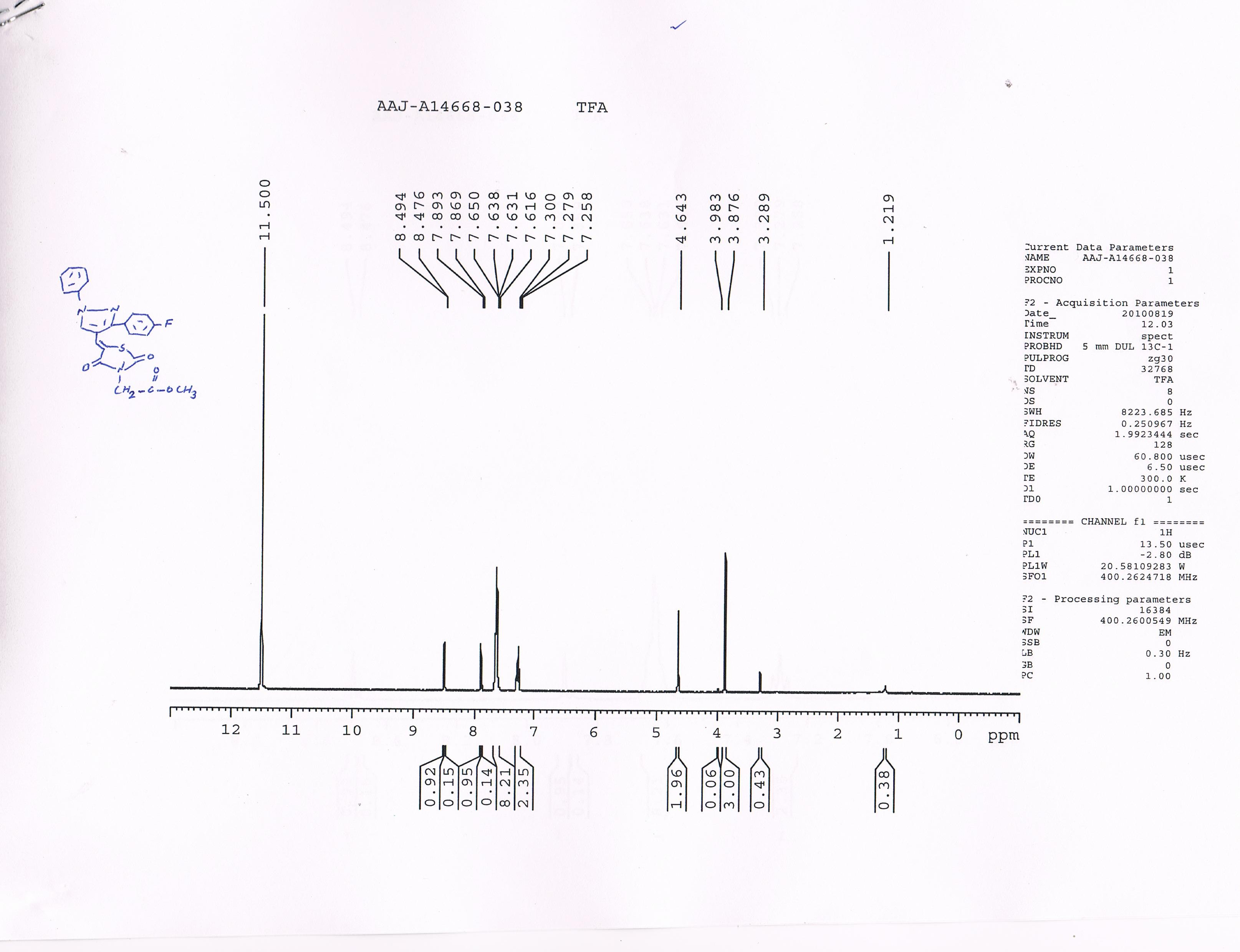

Supplement: Additional file 13 — 1H NMR Spectra .(5e); 1H NMR of methyl 2-((Z)-5-((3-(4-fluorophenyl)-1-phenyl-1H-pyrazol-4-yl)methylene)-2, 4-dioxothiazolidin-3-yl)acetate [file 2191-2858-1-15-S13.JPEG]

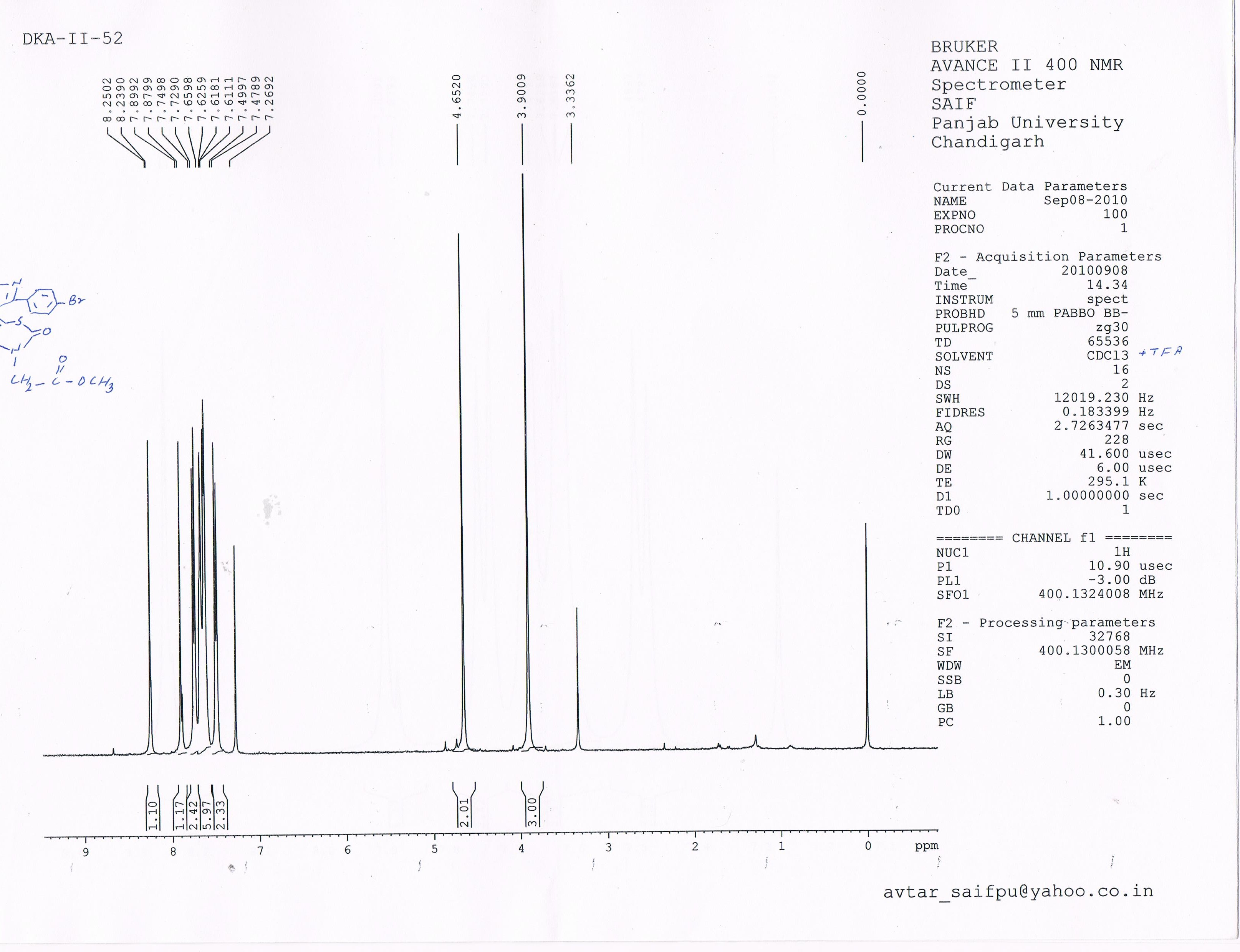

Supplement: Additional file 14 — 1H NMR Spectra .(5f); 1H NMR of methyl 2-((Z)-5-((3-(4-bromophenyl)-1-phenyl-1H-pyrazol-4-yl)methylene)-2, 4-dioxothiazolidin-3-yl)acetate [file 2191-2858-1-15-S14.JPEG]

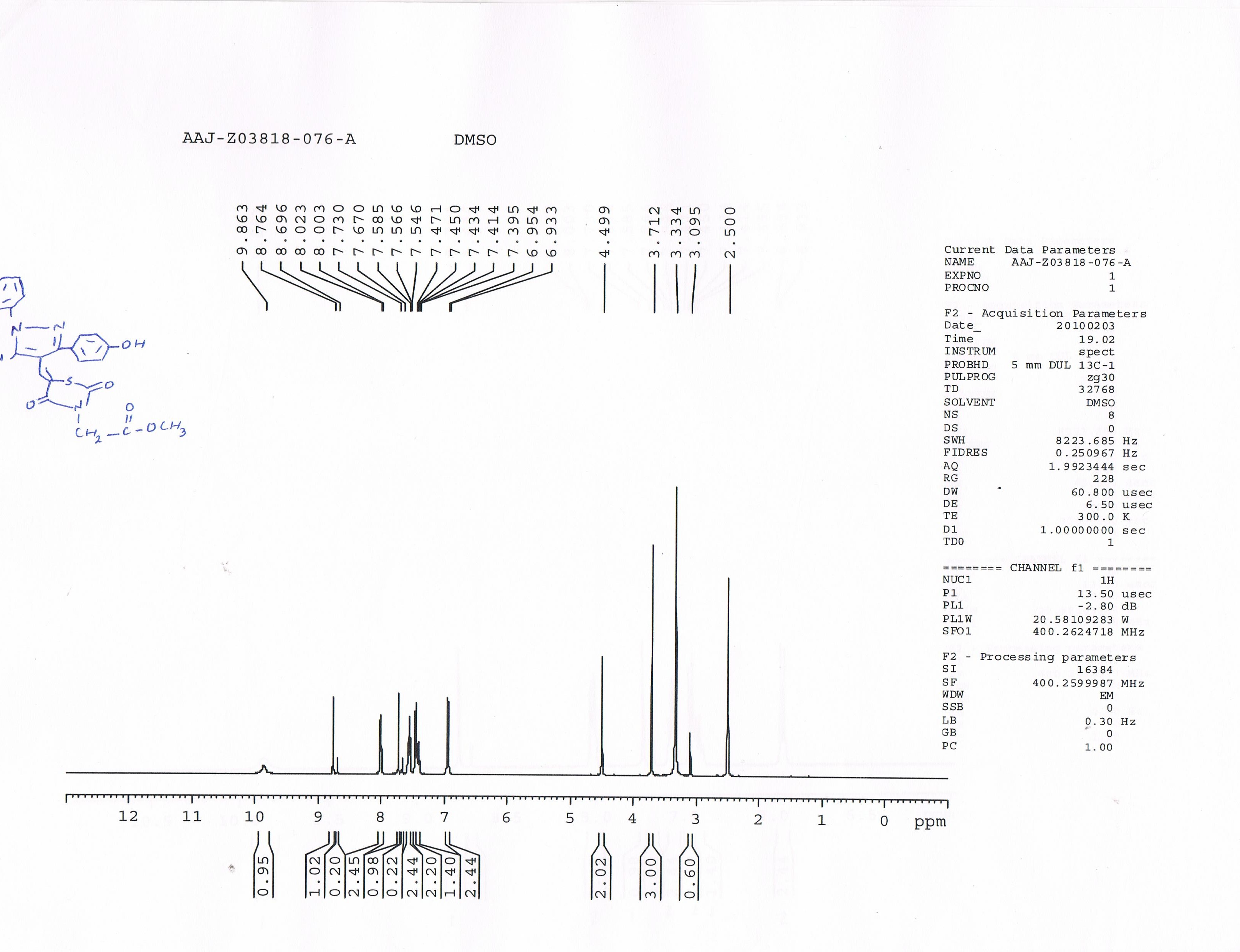

Supplement: Additional file 15 — 1H NMR Spectra .(5g); 1H NMR of methyl 2-((Z)-5-((3-(4-hydroxyphenyl)-1-phenyl-1H-pyrazol-4-yl)methylene)-2, 4-dioxothiazolidin-3-yl)acetate [file 2191-2858-1-15-S15.JPEG]

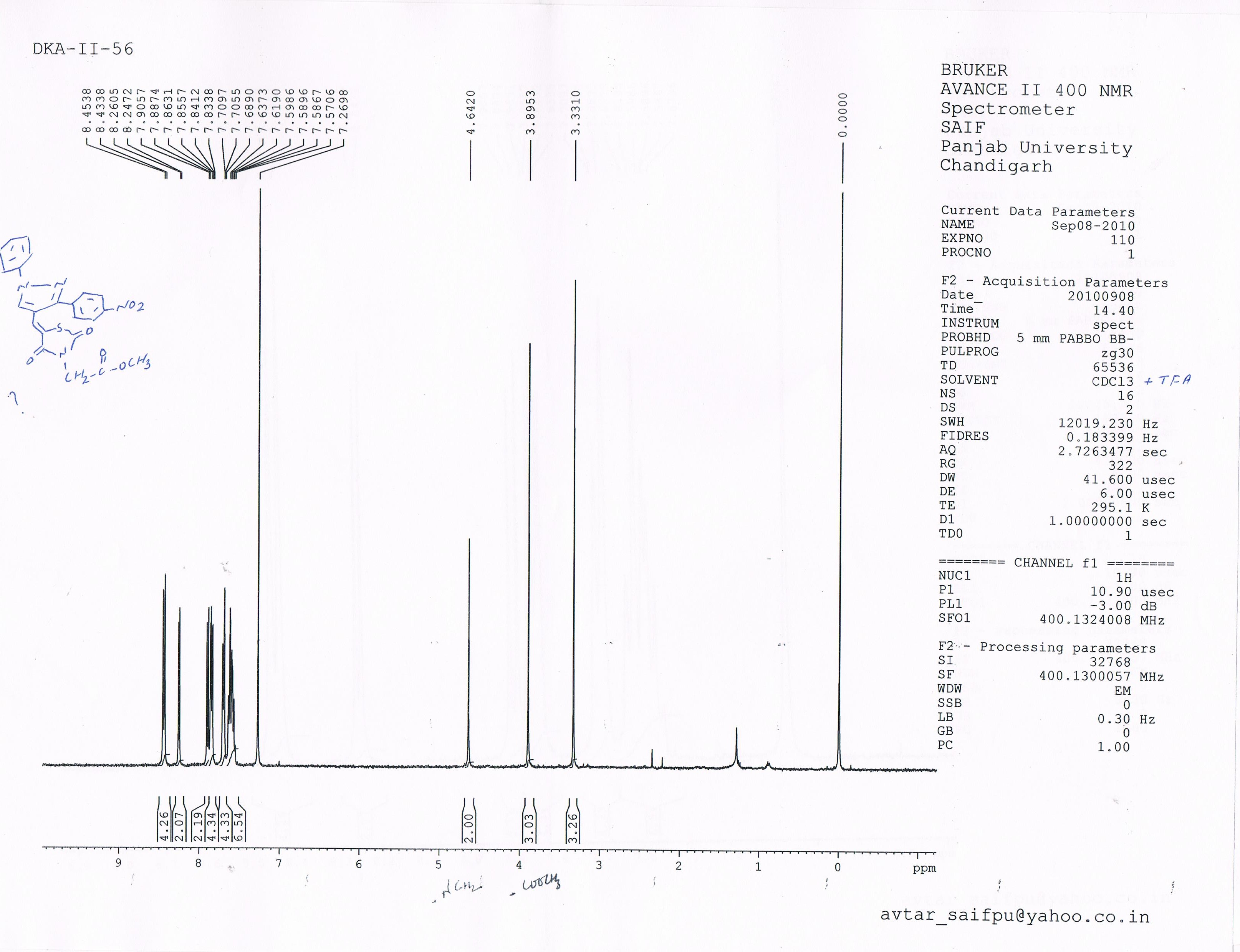

Supplement: Additional file 16 — 1H NMR Spectra .(5h); 1H NMR of methyl 2-((Z)-5-((3-(4-nitrophenyl)-1-phenyl-1H-pyrazol-4-yl)methylene)-2, 4-dioxothiazolidin-3-yl)acetate [file 2191-2858-1-15-S16.JPEG]

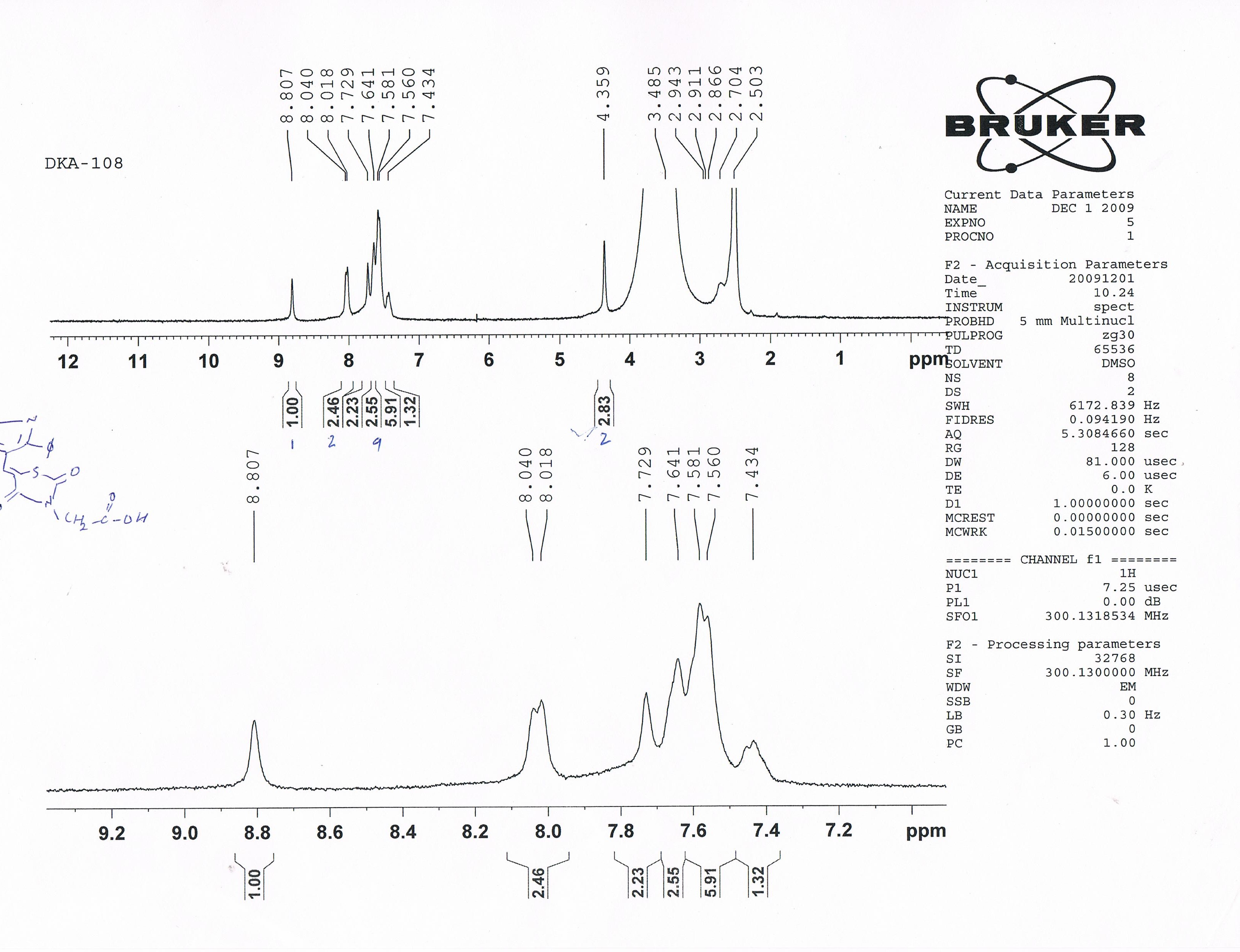

Supplement: Additional file 17 — 1H NMR Spectra .(6a); 1H NMR of 2-((Z)-2, 4-dioxo-5-((1, 3-diphenyl-1H-pyrazol-4-yl)methylene)thiazolidin-3-yl)acetic acid [file 2191-2858-1-15-S17.JPEG]

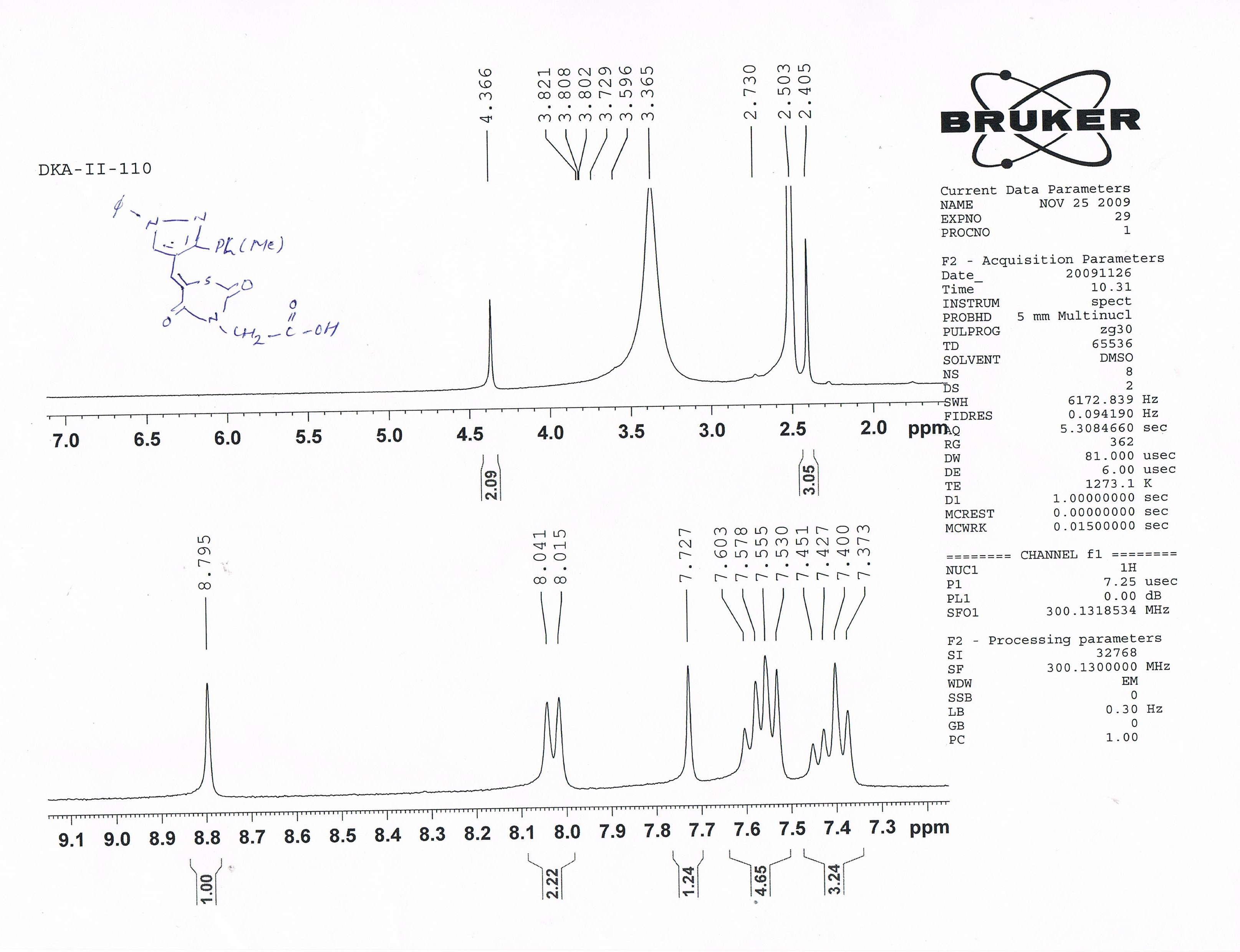

Supplement: Additional file 18 — 1H NMR Spectra .(6b); 1H NMR of 2-((Z)-2, 4-dioxo-5-((1-phenyl-3-p-tolyl-1H-pyrazol-4-yl)methylene)thiazolidin-3-yl)acetic acid [file 2191-2858-1-15-S18.JPEG]

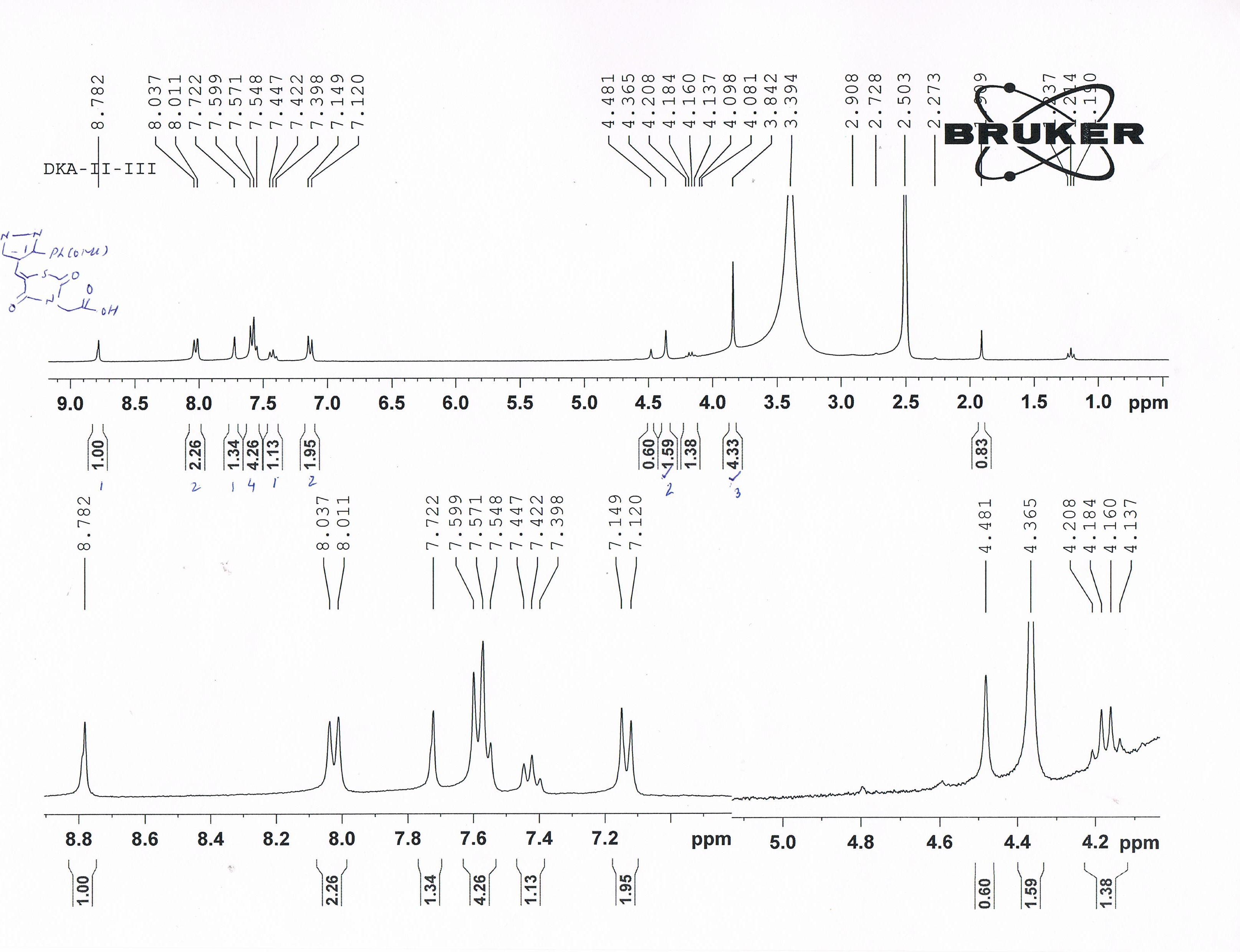

Supplement: Additional file 19 — 1H NMR Spectra .(6c); 1H NMR of 2-((Z)-5-((3-(4-methoxyphenyl)-1-phenyl-1H-pyrazol-4-yl)methylene)-2, 4-dioxothiazolidin-3-yl)acetic acid [file 2191-2858-1-15-S19.JPEG]

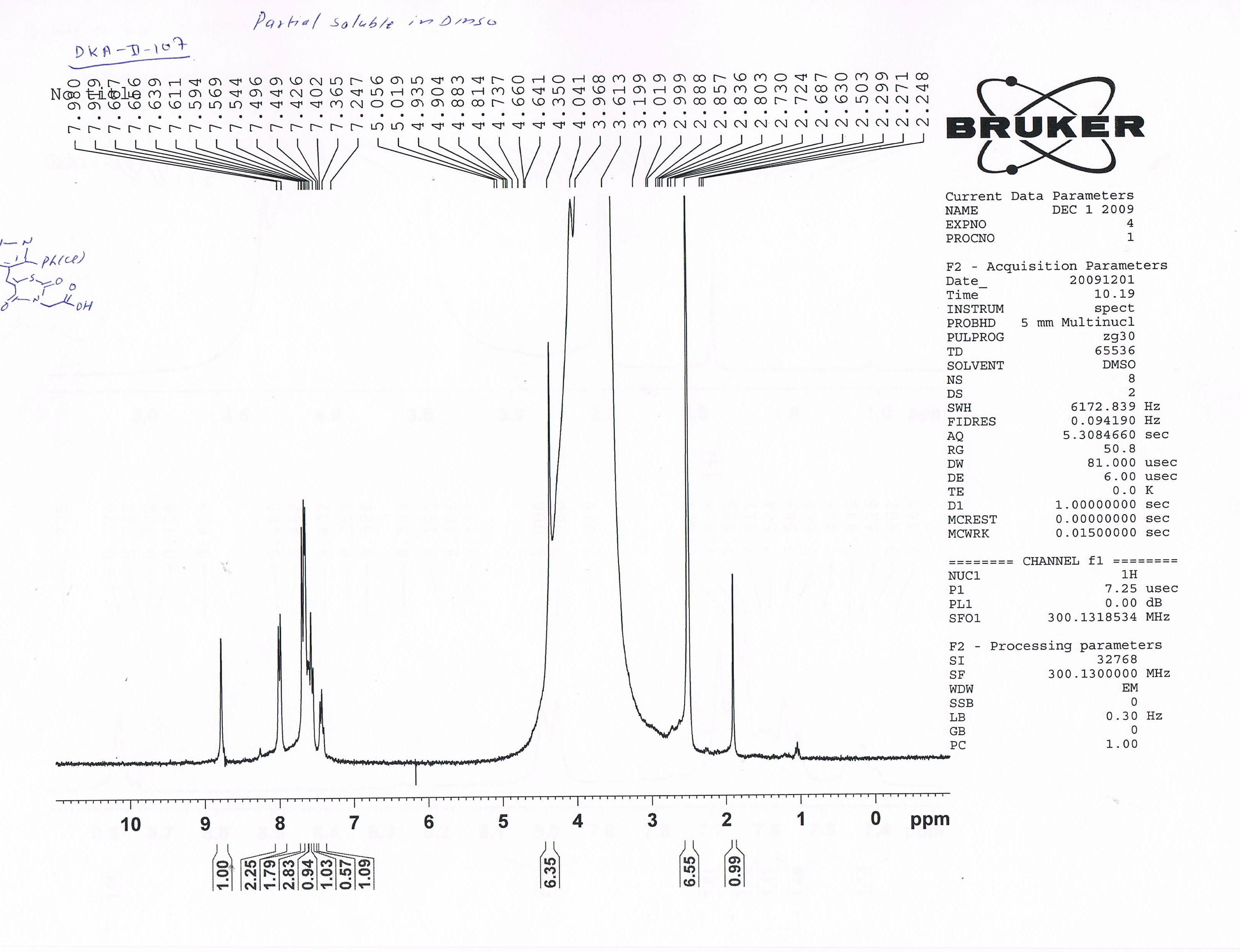

Supplement: Additional file 20 — 1H NMR Spectra .(6d); 1H NMR of 2-((Z)-5-((3-(4-chlorophenyl)-1-phenyl-1H-pyrazol-4-yl)methylene)-2, 4-dioxothiazolidin-3-yl)acetic acid [file 2191-2858-1-15-S20.JPEG]

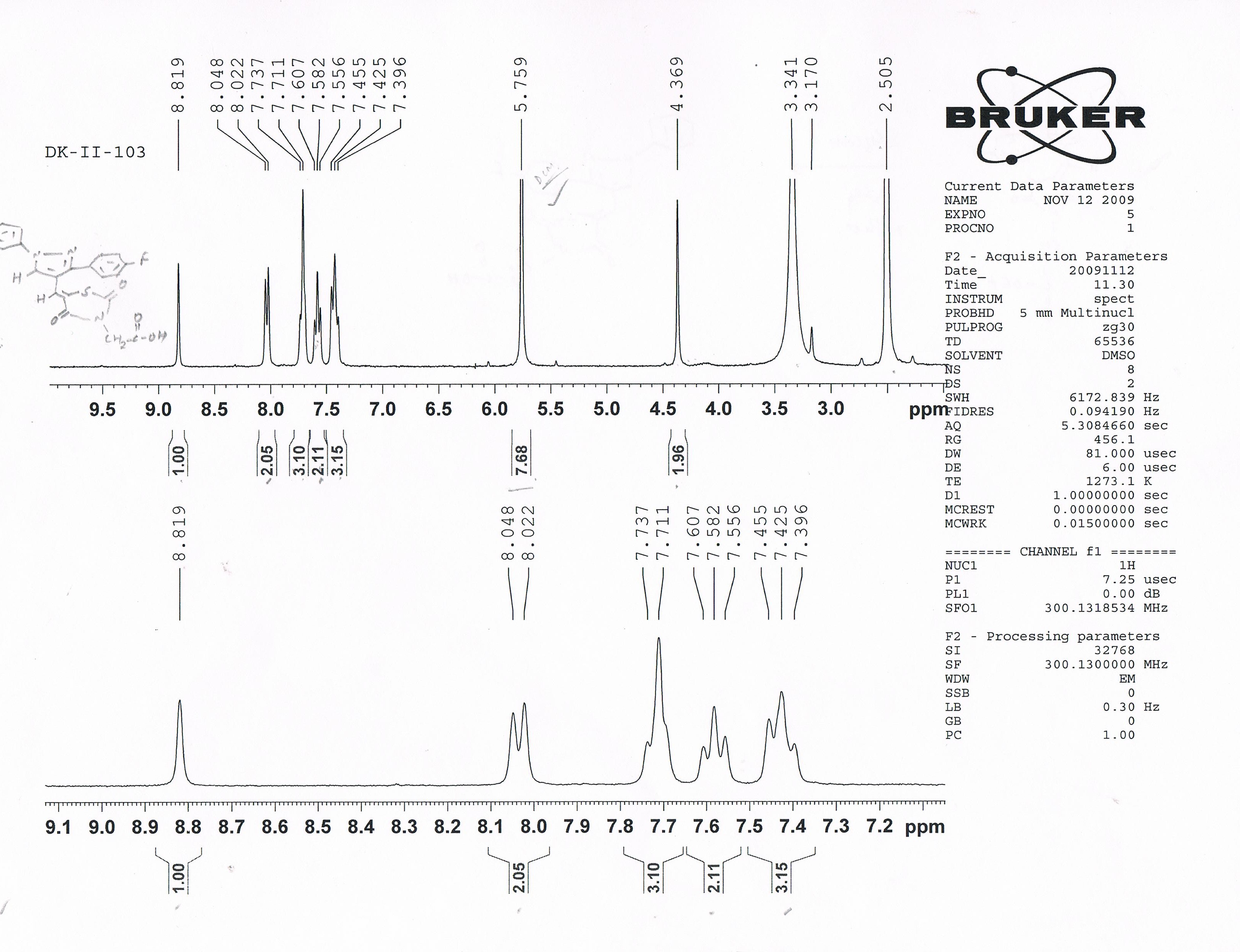

Supplement: Additional file 21 — 1H NMR Spectra .(6e); 1H NMR of 2-((Z)-5-((3-(4-fluorophenyl)-1-phenyl-1H-pyrazol-4-yl)methylene)-2, 4-dioxothiazolidin-3-yl)acetic acid [file 2191-2858-1-15-S21.JPEG]

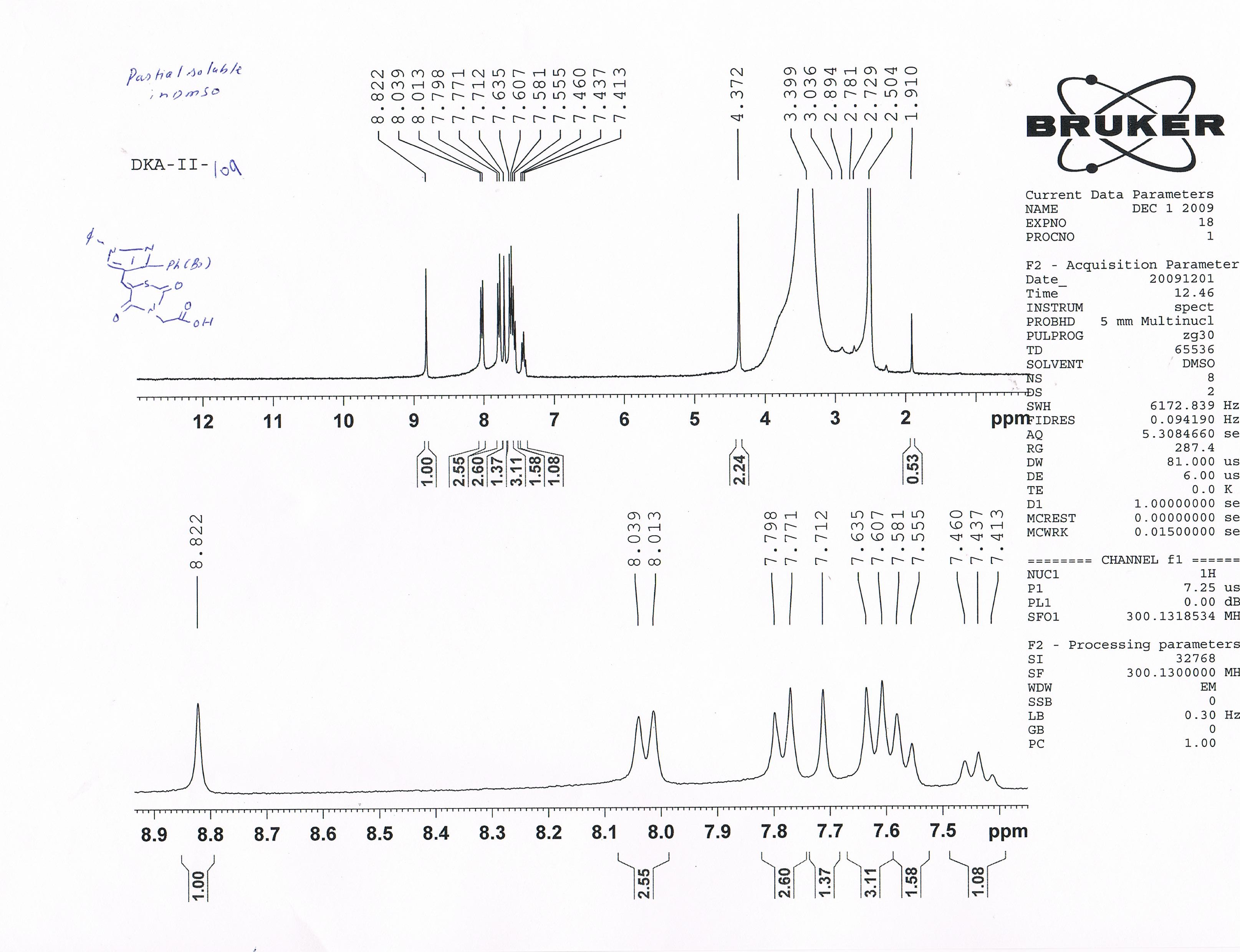

Supplement: Additional file 22 — 1H NMR Spectra .(6f); 1H NMR of 2-((Z)-5-((3-(4-bromophenyl)-1-phenyl-1H-pyrazol-4-yl)methylene)-2, 4-dioxothiazolidin-3-yl)acetic acid [file 2191-2858-1-15-S22.JPEG]

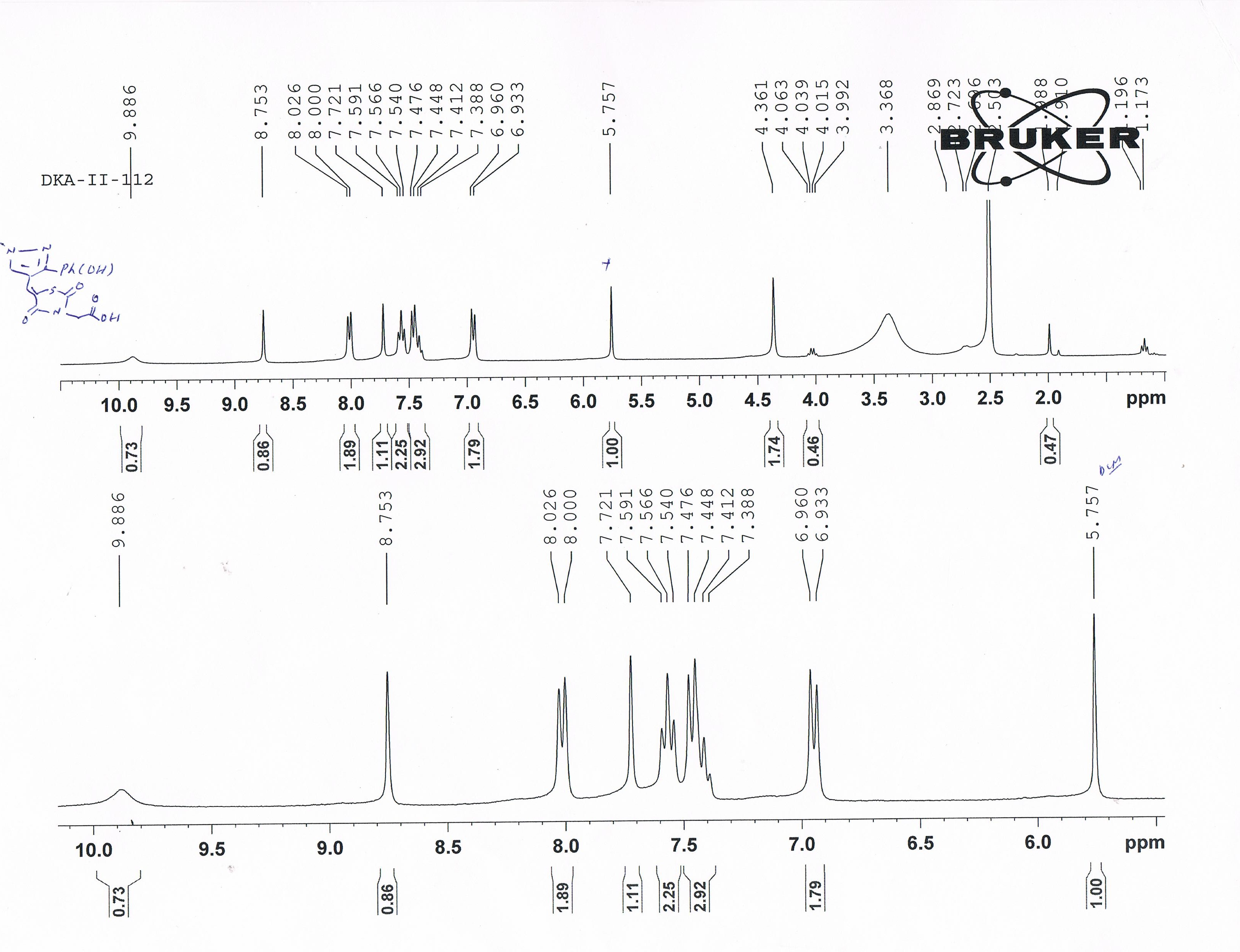

Supplement: Additional file 23 — 1H NMR Spectra .(6g); 1H NMR of 2-((Z)-5-((3-(4-hydroxyphenyl)-1-phenyl-1H-pyrazol-4-yl)methylene)-2, 4-dioxothiazolidin-3-yl)acetic acid [file 2191-2858-1-15-S23.JPEG]

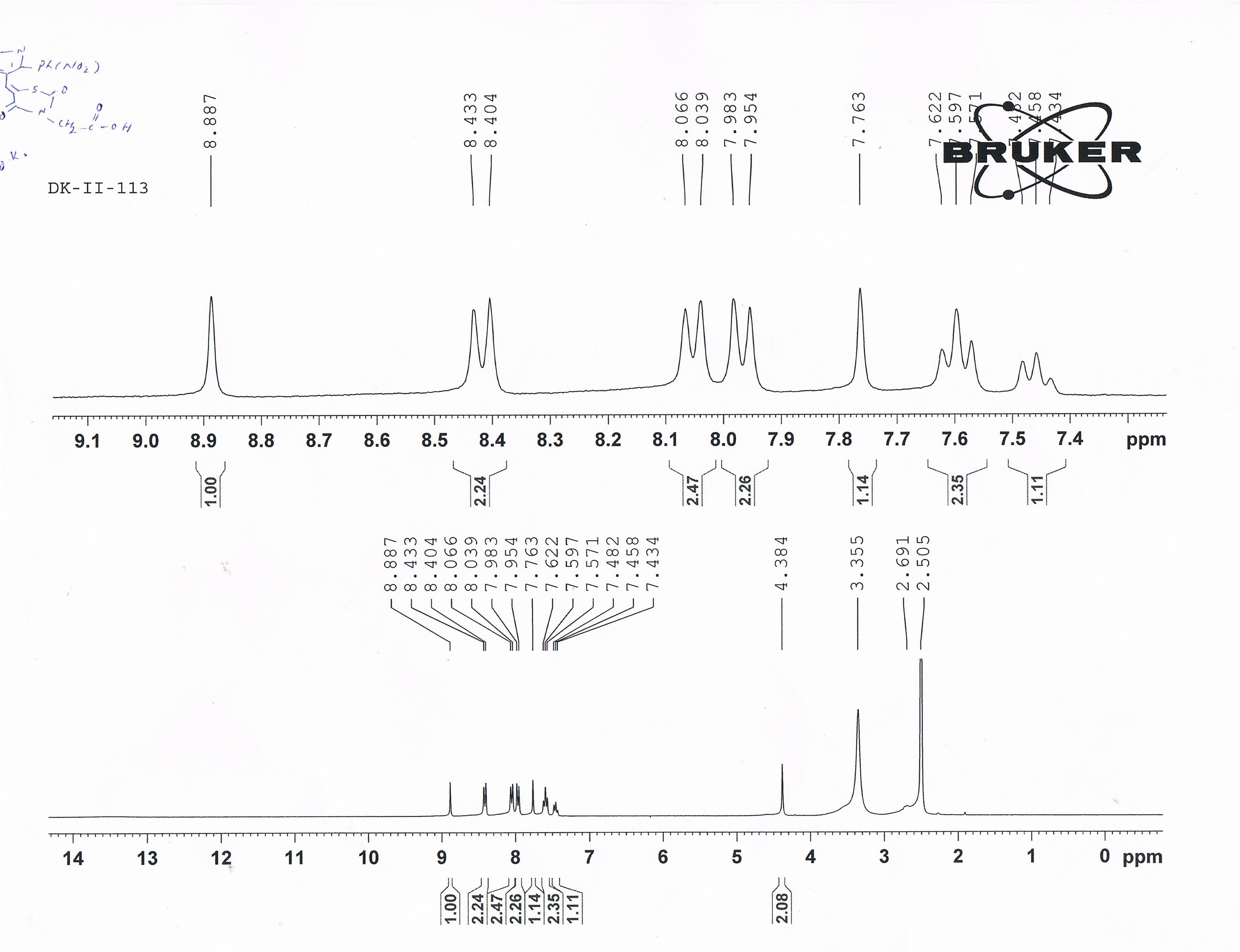

Supplement: Additional file 24 — 1H NMR Spectra .(6h); 1H NMR of 2-((Z)-5-((3-(4-nitrophenyl)-1-phenyl-1H-pyrazol-4-yl)methylene)-2, 4-dioxothiazolidin-3-yl)acetic acid [file 2191-2858-1-15-S24.JPEG]
